# Structural variants in 3000 rice genomes

**DOI:** 10.1101/gr.241240.118

**Published:** 2019-05

**Authors:** Roven Rommel Fuentes, Dmytro Chebotarov, Jorge Duitama, Sean Smith, Juan Fernando De la Hoz, Marghoob Mohiyuddin, Rod A. Wing, Kenneth L. McNally, Tatiana Tatarinova, Andrey Grigoriev, Ramil Mauleon, Nickolai Alexandrov

**Affiliations:** 1 International Rice Research Institute, Laguna 4031, Philippines;; 2 Bioinformatics Group, Wageningen University and Research, 6708 PB Wageningen, the Netherlands;; 3 Systems and Computing Engineering Department, Universidad de Los Andes, Bogotá 111711, Colombia;; 4 Agrobiodiversity Research Area, International Center for Tropical Agriculture (CIAT), Cali 6713, Colombia;; 5 Biology Department, Center for Computational and Integrative Biology, Rutgers University, Camden, New Jersey 08102, USA;; 6 Roche Sequencing Solutions, Belmont, California 94002, USA;; 7 Arizona Genomics Institute, University of Arizona, Tucson, Arizona 85721, USA;; 8 King Abdullah University of Science and Technology, Thuwal 23955, Saudi Arabia;; 9 Department of Biology, University of La Verne, La Verne, California 91750, USA;; 10 Vavilov Institute of General Genetics, Moscow 119333, Russia;; 11 A.A. Kharkevich Institute for Information Transmission Problems, Russian Academy of Sciences, Moscow 127051, Russia;; 12 Laboratory of Forest Genomics, Siberian Federal University, Krasnoyarsk 660041, Russia

## Abstract

Investigation of large structural variants (SVs) is a challenging yet important task in understanding trait differences in highly repetitive genomes. Combining different bioinformatic approaches for SV detection, we analyzed whole-genome sequencing data from 3000 rice genomes and identified 63 million individual SV calls that grouped into 1.5 million allelic variants. We found enrichment of long SVs in promoters and an excess of shorter variants in 5′ UTRs. Across the rice genomes, we identified regions of high SV frequency enriched in stress response genes. We demonstrated how SVs may help in finding causative variants in genome-wide association analysis. These new insights into rice genome biology are valuable for understanding the effects SVs have on gene function, with the prospect of identifying novel agronomically important alleles that can be utilized to improve cultivated rice.

Genomics accelerates biotechnological discoveries and advances in crops and livestock, particularly by identifying genetic markers and characterizing molecular mechanisms behind desirable traits that will aid in generating new varieties through marker-assisted breeding and genome editing. This is of particular importance for rice, which needs an estimated 26% increase in yield to meet the global demand by the year 2030 under constraints such as less arable land, less water, and severe environmental stresses due to climate change (Seck et al. 2012).

To help address this yield gap, we intend to catalog all natural variation that exists in cultivated and wild rice and utilize that information to identify genes and genomic regions that can be used to drive the next generation of super crops. As an initial foray, we resequenced 3010 rice genomes (3K RG) and discovered ∼20 million SNPs upon alignment to the Nipponbare reference sequence (Alexandrov et al. 2014; The 3000 rice genomes project 2014). Further efforts expanded this database by integrating short insertions and deletions (indels) into the data set (Mansueto et al. 2017). Recent studies, however, reveal that single-nucleotide polymorphisms (SNPs) do not capture the entire spectrum of variations contributing to phenotypic differences, and structural variants also play an important role (Saxena et al. 2014; Francia et al. 2015).

Detection and characterization of structural variants (SVs) has revolutionized the understanding of the landscape of genomic variation in different species. A structural variant is commonly defined as a change in the genome (relative to a reference genome) that has a different copy number (i.e., gain, loss, deletion), orientation, or chromosomal location (Medvedev et al. 2009; Escaramís et al. 2015). In human genomes, structural variants account for more varying base pairs than SNPs (Alkan et al. 2011; Baker 2012; Sudmant et al. 2015); yet, in plants, studies of SVs are still limited (Saxena et al. 2014). Although less common than SNPs, structural variants have a greater potential to impact function due to their larger size and the possibility of altering gene structure, dosage, or location (Layer et al. 2014).

After the discovery that structural genomic variation in human genomes is common, more SV studies were initiated in other species, from the agriculturally important (Swanson-Wagner et al. 2010) to extinct ones (Smith et al. 2017b). However, identification of SVs generally has lagged behind finding single-nucleotide variants due to the lack of high-quality reference genomes (Escaramís et al. 2015) and robust methods, both of which are needed to discover and genotype SVs. In plants, structural variants are not recognized as polymorphisms affecting individual plants but as differentiating elements between cultivars/accessions of one species (Francia et al. 2015). Maize became the first plant species to be extensively interrogated to discover hundreds of SVs. Although the number of SVs detected was later found to be an underestimate, the high level of SVs in maize was unprecedented among higher eukaryotes (Żmieńko et al. 2014). Another large plant genome sequencing initiative started in 2008 developed a catalog of genetic variation in 1135 *Arabidopsis* accessions (The 1001 Genomes Consortium 2016).

Several studies in plants have already shown the association between structural variants and plant phenotypes (Żmieńko et al. 2014). For example, the increased copy number of *Vrn-A1* and *Ppd-B1* genes in wheat causes late flowering and early flowering, respectively (Würschum et al. 2015). Furthermore, a specific tandem duplication in wheat that covers the *Rht-D1b* gene results in a >70% reduction in plant height (Li et al. 2012). SVs have also been linked to stress tolerance phenotypes in crop plants such as boron tolerance in barley (Sutton et al. 2007) and nematode resistance in soybean (Cook et al. 2012).

Previous studies on rice (*O. sativa*) have identified structural variants by comparison of rice genome to its closest relatives in genus *Oryza* (Hurwitz et al. 2010) and between representatives of its major subgroups (Schatz et al. 2014) and elucidated association between structural variants and rice phenotypes using multiple rice accessions (Xu et al. 2012; Duitama et al. 2015). Examples of SVs affecting rice traits include the 17.1-kb tandem duplication at the *GL7* locus (Wang et al. 2015) that increases grain length, the 1.2-kb deletion in *qSW5* that alters grain width (Shomura et al. 2008), the 833-bp deletion that causes dwarf phenotypes and smaller grains (Ashikari et al. 1999), and the 10-bp deletion that results in slender grains (Wang et al. 2012). Recently, an extensive study on genomic variants including 90,000 SVs larger than 100 bp in the 3K RG was published, relying on a single SV caller (Wang et al. 2018) applied to a subset of samples with high coverage.

The mutational mechanism for structural variants formation includes nonallelic homologous recombination, nonhomologous end-joining (NHEJ), shrinking or expansion of variable number tandem repeats, and transposable element insertion (TEI) (Lam et al. 2010; Yi and Ju 2018). In the human genome, NHEJ and TEI are the major mechanisms for SV formation (Lam et al. 2010; Yang et al. 2013).

Structural variants can be classified in the following types: deletions, insertions, duplications (tandem and interspersed), inversions, and translocations. There are five general strategies to detect SVs based on analysis of data from high-throughput sequencing data using short reads: paired-end mapping (RP) (Chen et al. 2009; Sindi et al. 2009), split-read mapping (SR) (Schröder et al. 2014), read depth (RD) (Abyzov et al. 2011; Duitama et al. 2014; Smith et al. 2015), de novo assembly (AS) (Narzisi et al. 2014; Rizk et al. 2014; Yang et al. 2015), and a combination of the preceding approaches (CB) (Ye et al. 2009; Rausch et al. 2012; Layer et al. 2014; Mohiyuddin et al. 2015; Smith et al. 2017a). Each of these strategies has different strengths and weaknesses in detection, depending on variant type, sequence length, and reference genome quality and complexity; hence, applying complementary methods and combining results can overcome some of the limitations inherent to these different approaches (Alkan et al. 2011).

Despite the development of many SV callers, SV discovery remains challenging due to the complexity of some structural variant events and their occurrence in repetitive regions (Sudmant et al. 2015). For example, 45% of the rice genome consists of repetitive sequences (Ouyang and Buell 2004), complicating read mapping and reducing accuracy of breakpoint predictions. Aside from the performance of the callers, the nature of the data set greatly influences the quality of prediction. Many studies suggest that sensitivity, specificity, and breakpoint accuracy are dependent on read length, insert size, and physical coverage (Alkan et al. 2011). Because the average sequence coverage of the 3K RG data set is 14× depth, the use of one single method for SV detection may result in a high error rate.

In this study, we combined multiple approaches and developed a robust SV prediction pipeline to identify more than 63 million structural variants grouped into 1.5 million SV events across 3000 rice genomes and performed further analyses to confirm their accuracy. This set of SVs represents an important public resource cataloging genome variation across the main rice varieties and provides new insights for the discovery of genes related to different traits and for studying the possible roles of structural variants in rice.

## Results

### SV clusters number 1.5 million within *O. sativa*

**Figure 1.**
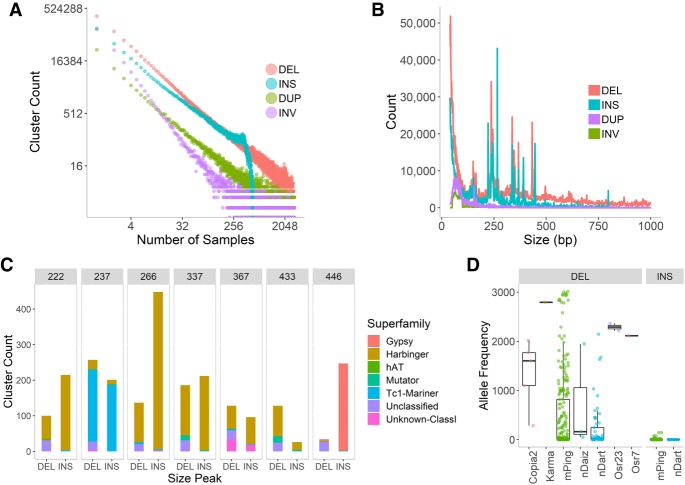
Distribution and classification of SVs. (*A*) Frequency of observations per SV cluster. Only 562 high-coverage samples were used for insertion detection. (*B*) Distribution of variant sizes by SV type. (*C*) Classification of variants in each peak (cluster frequency > 10 samples). (*D*) Frequencies of events with 98% sequence identity to known or potentially active TEs in rice.

Based on the benchmark of 10 diverse SV-finding algorithms (Supplemental Methods; Supplemental Fig. S1; Supplemental Table S2), we built a custom SV calling pipeline and used it to detect deletions, insertions, tandem duplication, and inversions on the 3K RG data set and alignment files (https://aws.amazon.com/public-datasets/3000-rice-genome/). Instead of relying on a single caller, we combined multiple variant callers with the best sensitivity and precision across different sizes and types of SVs. We identified a total of 63,441,115 SV calls (Table 1) across the 3K RG data set and grouped them into 1.5 million SVs clusters or events. The clusters were defined by grouping together SV calls in different samples that are likely to correspond to single evolutionary events. This grouping was based on the similarity in sizes and positions of SVs, with an average distance between breakpoints on either side of each cluster of 2.2% of the respective SV length (Supplemental Fig. S2). The frequency distribution for each type of SV follows the power law (Fig. 1A), consistent with expectation from the neutral theory of evolution (Fu 1995). Compared to SVs discovered by Wang et al. (2018), our data set covered 80.5% of their detected SV sites (Supplemental Fig. S3) within the same subset of samples with high coverage; however, we also report insertions and variants smaller than 100 bp, applied more stringent clustering criteria, and used all 3K RG samples for SV detection.

**Table 1.**
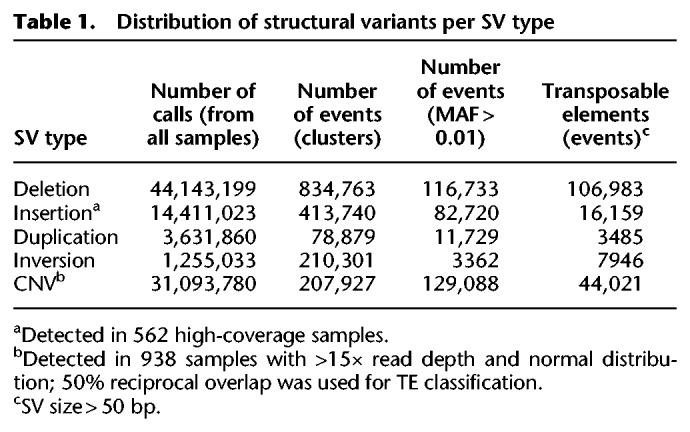
Distribution of structural variants per SV type

To further validate SVs detected by our pipeline, we compared the reference genome of Nipponbare (IRGSP 1.0) (Kawahara et al. 2013) with the published genome of N 22 (Pacific Biosciences [PacBio] assembly) (Stein et al. 2018) by visually inspecting predicted variants using a dot plot display (Krumsiek et al. 2007) of the two genomes aligned against each other (Supplemental Fig. S17) and calculated false positive rates (FPR) and false negative rates (FNR) for random variants predicted in the sample CX368, an N 22 accession. The FPR for deletions is 14%, whereas duplications and inversions have much higher FPRs, 40% and 75%, respectively (Supplemental Table S4). Predicted false positive rates of the pipeline across different types of variants compare favorably with the performance of individual tools; for example, see extensive benchmarking of several leading SV callers on human genomes from Illumina sequencing (Smith et al. 2017b). The false negative rate for detecting deletions is ∼40%, consistent with the use of multiple callers whereby caller-specific SVs are often discarded and dependent on the quality of the sequences. The number of detected inversions and tandem duplications is very low compared to the other types. Although this observation can reflect a limitation of the detection pipeline, it is consistent with studies in other organisms (Quinlan et al. 2010). Due to the lower coverage of sample CX368 and its non-normal read depth distribution (Supplemental Figure S18a), we were not surprised by somewhat higher FPR and FNR, and we could not validate insertions and CNVs predicted based on read depth following this procedure (for precision of CNV, see Supplemental Figure S18b).

### Transposable elements

Transposable elements play a major role in creating SVs. Among the 8.7% SV events (17.3% SVs calls) that have 80% reciprocal overlap (at least 80% of the TE is covered by SV, and at least 80% of the SV is covered by TE) with known transposable elements (TEs) in Nipponbare annotated in the RiTE database (Copetti et al. 2015), the Harbinger superfamily had the largest SV event contribution, followed by the Tc1-Mariner and Mutator TE families (Supplemental Table S1). Of the remaining clusters, 42.5% also overlap on at least 50% of their length with the TE and repetitive regions, comprising ∼45% of the rice genome (Ouyang and Buell 2004), but with lower intersection percentages.

Most peaks in the distribution of SV sizes (Fig. 1B) match TEs defined in the RiTE database with major peaks associated with events related to the Tc1-mariner (DTT), Harbinger (DTH), Mutator (DTM), Gypsy (RLG), and SINE (RSU) families of transposable elements (Fig. 1C). Peaks at 237, 433, and 466 bp consisted of the OsT38 family of Tc1-mariner (Lu et al. 2012) elements, mPing elements (Jiang et al. 2003; Naito et al. 2014), and the long terminal repeat (LTR) of the gypsy-type retrotransposon RIRE2 (Ohtsubo et al. 1999), respectively. Last, we found that 87.6% of the 155-bp duplication peak is composed of centromeric repeats (SRC).

Using the MEME Suite (Bailey et al. 2009), we analyzed the deletion sequences corresponding to the 237- to 238-bp peaks and found that 81.1% have rice-specific 95-bp terminal inverted repeats (TIR). Insertions of 237 and 238 bp were found to have the same 95 bp TIR and were classified as Tc1-mariner elements, one of the superfamilies that generates the majority of the miniature inverted-repeat transposable elements (MITEs) in rice (Han et al. 2013). Of the deletions with TIR, 90% (40,212) belong to 191 (28%) clusters with a frequency above 0.1. We identified 730 genes that have insertion/deletion of MITEs in the 3′ UTRs, which may result in the translational repression of the gene as shown by Shen et al. (2017). Their conserved lengths are consistent with observations of the OsT38 family of Tc1-mariner (Lu et al. 2012).

Other known active transposable elements also matched several events (Fig. 1D). Supplemental Figure S19 shows more TE families that matched SVs and that mPing inserts preferentially in introns, whereas nDart tends to insert in 5′ UTRs.

Supplemental Table S1 presents statistics of transposable and repeat elements (size >50 bp) among the detected structural variants. Even after aggregation of insertions and duplications, detected events are significantly rarer than deletion events: Counting SV events, the ratio of the number of deletions to the combined number of insertions and duplications is 5.45. There are several reasons for this imbalance. First, when sequences of any two genomes are compared, deletions in one genome are detected as insertions in the other. Because we do not have the ancestral genome, unaffected by expansion of TE, we must compare three thousand genomes of cultivated rice to the Nipponbare reference. All insertions of transposable elements in the evolution of *Japonica* rice or even particular to Nipponbare will be predicted as deletions in other accessions in a reference-based analysis. Second, insertion and other events (especially longer ones) in accessions different from Nipponbare are much harder to detect using short NGS reads compared to the deletion events. Hence, a large proportion of nondeletion events, therefore, may go undetected. Comparison of length of different TEs supports the importance of this factor. A typical LINE element can be as long as 6000 base pairs, whereas LTRs range from 100 to 5000 base pairs. Supplemental Table S1 shows that predicted LINE element deletions are almost 100 times more common compared to predicted insertions, whereas for various subclasses of LTR, the ratio can be as small as 1.5.

This tendency of excess deletions occurs for both retrotransposons (Class I; copy and paste) and DNA transposons (Class II; cut and paste). Overall, there are more deletion events for Class II transposons. This is a combined methodological and biological effect that can be illustrated by the following thought experiment. Assuming that there are copy-and-paste TE events in each of the 3K rice genomes. When projected onto the reference genome, they should be manifested as insertions. Because insertions are hard to detect using NGS, many of them go undetected. When a cut-and-paste event occurs in the same genomes, the “cut” site is seen as a deletion event, and the “paste” site can be either detected or missed. This leads to a greater number of deletions of Class II TEs (Supplemental Table S1). The copy-and-paste mechanism ensures a relatively higher number of insertions for Class I TEs. Our observations support both of these hypotheses in which the ratio of the number of detected deletions to insertions and duplications for Class I is 2.36, and for Class II (cut-and-paste mechanism), it is 6.77.

To normalize the described imbalance effect produced by the methodology, for each type of SV instead of raw numbers, we compared the distribution (percentage) of events intersecting each TE family with the general distribution of TEs annotated in the rice reference genome. If SVs are randomly distributed, both distributions should be similar. For CNV and tandem duplications, we identify mostly Gypsy and Copia Class I elements (Supplemental Data, sheet “TE SVs”). Copia elements are more enriched in duplication (17.25%) than in deletion CNVs (10.46%). For the Class II elements, CNVs show enrichment for CATCA elements, probably because they are longer on average (860 bp) than other Class II elements. The Helitron family appears to be enriched in deletion CNVs (7,18%). Deletions, insertions, and inversions found by RP approaches are comprised mostly of Harbinger, Mutator, and Mariner Class II elements. Despite the larger numbers, percentages of deletions in Class I elements are similar to the general percentages of these elements. In contrast, Gypsy elements are enriched in insertions (17.15%) and inversions (12.52%). Cluster sizes for deletions related to Copia and Gypsy elements are much larger on average than clusters for other elements. This suggests that many of these events may be true insertions in *Japonica*. Despite the shortcomings of each method to detect SVs, our data agree with the expected footprints of historical activity of Class I and Class II transposable elements in contributing to variety-specific (or type-specific) differences in genome structure.

### Population structure derived from SV calls

**Figure 2.**
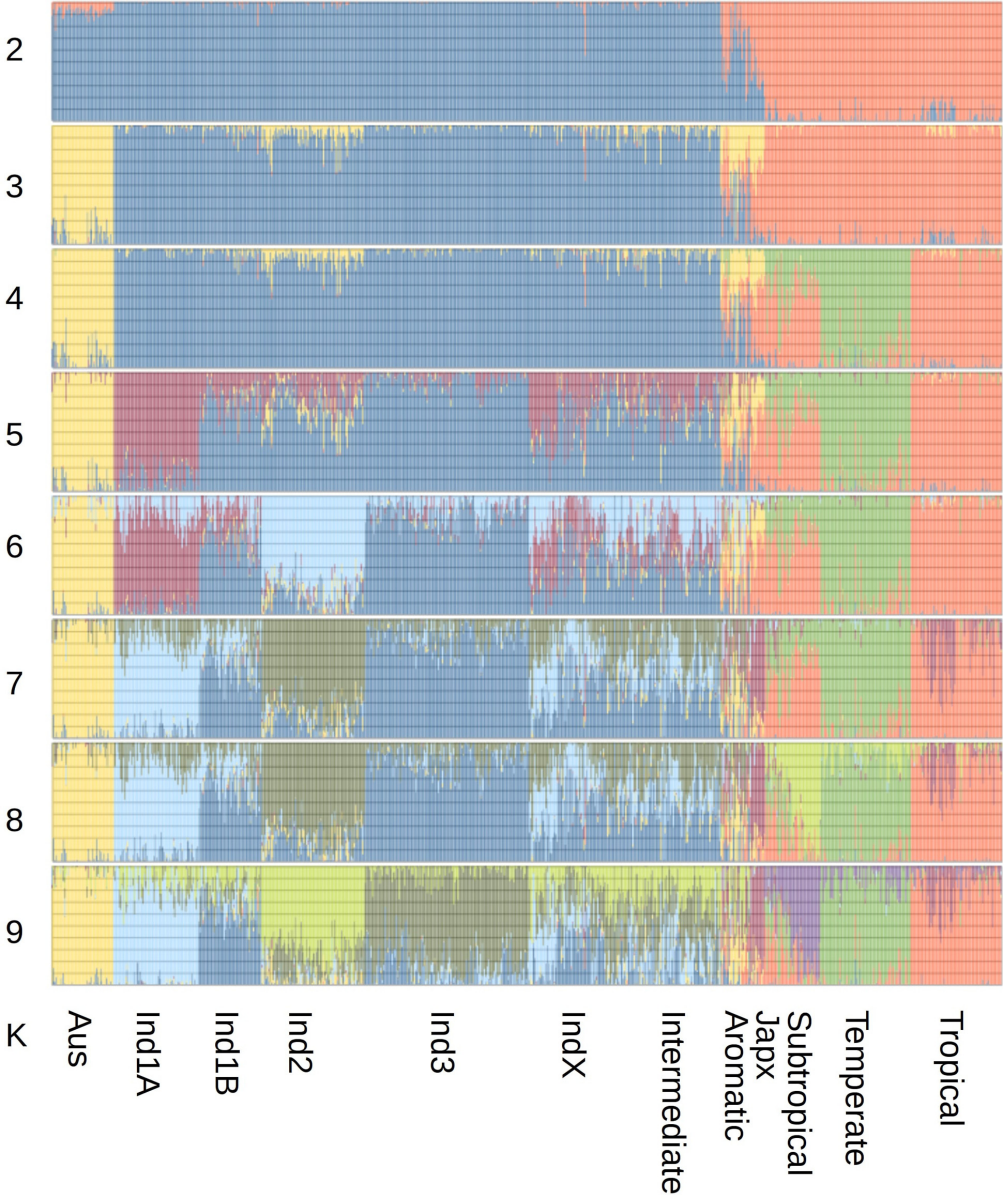
Structure analysis based on selected CNVs and assuming *K* = [2, …, 9] subpopulations.

To validate the catalog of SVs described in this study, we selected different types of variants and performed population structure analyses taking individual SV calls as alleles of genetic markers to verify whether this structure is consistent with that inferred from SNP markers. For the case of copy number variants (CNVs) genotype calls, we selected 7515 CNVs genotyped in at least 800 of 938 selected samples (for details, see Supplemental Methods; Supplemental Fig. S13) and having the major allele in a maximum of 99% of the samples. Then, using the predicted copy number of these CNVs in each accession as alleles of genetic markers, we performed a population stratification analysis with Structure (Pritchard et al. 2000) and verified that the population structure derived from CNVs is consistent with that obtained from genome-wide SNPs (Wang et al. 2018). Figure 2 shows that indeed, CNV genotyping data can distinguish the three major rice subpopulations of: *Indica*, *circum*-*Aus*, and *Japonica*. At *K* = 4, *Japonica* is separated into temperate and tropical types. At *K* = 5, the *Indica* group 1A emerges. From *K* = 6 to *K* = 9, *Indica* is further divided in the groups *Indica* 1A, 1B, 2, and 3, whereas *Japonica* is divided in temperate, tropical, subtropical, and admixed *Japonica* types. All of these groups are consistent with the clustering derived from SNP markers. We also tried to reconstruct population structure from 2839 CNVs genotyped in at least 2000 of the 3023 samples, located in nonrepetitive regions of the genome that have the major allele in at most 80% of the samples. In this case, the main populations could still be differentiated but the signal was less clear (Supplemental Fig. S14a). This was probably a result of the larger percentage of missing data in this data set (27.9%) compared to that of the data set shown in Figure 2 (9.02%) as well as the lower-quality predictions of CNVs for samples sequenced at low (<15×) average read depths.

We also conducted principal component analysis (PCA) on the deletion data set using all and high-coverage samples and found agreement with clustering defined by genome-wide SNP data (Wang et al. 2018). In particular, the first two principal components (PCs) separate major groups (Supplemental Fig. S4c), and PC 6 and 7 separate the *Indica* subgroups (Supplemental Fig. S4b,d).

### Distribution of SVs relative to gene models

**Figure 3.**
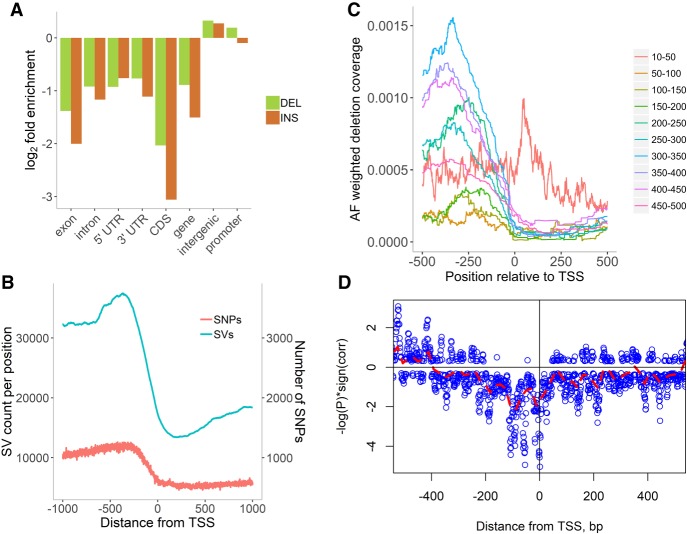
SVs in genome features. (*A*) Enrichment/depletion of deletions (green) and insertions (orange) in various genomic regions. As expected, genic regions have fewer SVs than intergenic ones, with CDSs and exons being the most conserved regions. (*B*) Distribution of deletion and insertion clusters near the transcription start site (TSS). Although the total number of SNPs is much larger than SV clusters, SVs affect more positions. The bump at about −366 bp just before the core promoter is explained by longer SVs associated with transposons. (*C*) Distribution of the number of deletions in the vicinities of start and end of transcription and translation (Supplemental Fig. S16). (*D*) *P*-values of the independence tests between predicted TFBS and deletions. Strong anti-correlation is observed at the TSS and ∼100 bp upstream. Distribution of *P*-values shows that in the core promoter area ([TSS-200, TSS]), deletions and TFBS are not independent.

About 74.6% of SV clusters lie in intergenic space, but only 5.8% intersect with exonic regions. Nevertheless, in the 3K RG data, we found that 72.6% of the gene models supported by full-length mRNA sequence overlap with SV clusters having MAF > 0.005, and 47.6% of their coding regions are affected by SVs. Structural variants occur more often in intergenic and promoter regions and are depleted in genic regions, especially in coding DNA sequence (CDS) (Fig. 3A). Figure 3B shows the similarity between distributions of SVs and SNPs (Tatarinova et al. 2016; Triska et al. 2017), where a higher density of variation was observed in the intergenic space. The excess of SV events upstream of core promoters is likely due to transposon-related SVs.

Figure 3C shows that there is a significant difference in distributions between short (<40 bp long) and long deletions. Short deletions peak in the 5′ UTR region, and long deletions are most frequent in the promoter region. Additionally, we examined short indels identified by the GATK (McKenna et al. 2010) pipeline and found that the peak in the 5′ UTR consists mostly of variants with sizes in multiples of 3 nt (Supplemental Fig. S5; Supplemental Fig. S15). Using the repeat finder SSRIT (Temnykh et al. 2001), we found that 42.4% of the small deletions within the region [transcription start site (TSS), TSS + 125] are simple sequence repeats (SSRs) with trimer or hexamer motifs (Supplemental Data, sheet “SSRs in UTRs”). Abundance of short indels in 5′ UTRs can be explained by high density of SSRs (mostly triplets) (Lawson and Zhang 2006), resulting in low sequence complexity (Supplemental Fig. S6).

To evaluate the effects of SVs on regulatory elements, for each position around the TSS, we performed a test for independence between the presence of transcription factor binding sites (TFBS) and deletions along the promoter sequence and found a significant negative correlation between the presence of TFBS and deletions near TSSs (Fig. 3D). In positions where *P*-values are close to 1 [log(P) close to zero], there is no mutual avoidance between TFBS and deletions. In the region [TSS-200, TSS + 50], there is significant mutual avoidance between TBFS and deletions, because the deletions in this area may be detrimental to the plant. Therefore, we hypothesize that presence of a functional TFBS in the core promoter decreases the chance of a structural variant to be retained.

### Association of SV-rich regions and stress response genes

Using 100-kb sliding windows with 50-kb overlaps, we computed the density of SNPs (Mansueto et al. 2017) and SVs across the rice genome and found that SNPs and SVs correlate (*r* = 0.52; *P* = 2.2 × 10^−16^) (Supplemental Fig. S7). We retrieved windows with SV counts of twice or more than the mean and performed an enrichment analysis using GO annotations. Supplemental Figure S8 shows highly similar distributions of SNPs and SVs across the genome and the colocalization of SV spikes and with enriched GO categories like “stress response” (Fisher's exact test; *P* = 2.8 × 10^−5^). At least 83.4% of the genes from the enriched categories overlapped a deletion (MAF > 0.005) (Supplemental Data, sheet “SV-Rich Genes”).

We also investigated possible functional roles of the genes affected by CNVs by selecting genes for which at least 80% of its genomic location was covered by a CNV used to perform structure analysis. Executing ontology term enrichment analysis by agriGO (Tian et al. 2017), we found that genes affected by CNVs were enriched for the biological processes of cell death and response to stress (Supplemental Fig. S14b). Enriched molecular functions include kinase activity and nucleotide binding (Supplemental Fig. S14c). This result is consistent with the previous analysis of Bai et al. (2016) on a more limited data set of deletions occurring in 50 accessions and suggests that copy number variation could play a role in the plant defense system.

### Known SVs at important loci

To test whether our pipeline detected known structural variants in rice, we examined a set of selected genes with known structural variants. An important gene in rice known as *GW5* was found to be associated with rice grain width and weight (Shomura et al. 2008). A study revealed that a deletion in *qSW5*, a QTL for seed width which contains *GW5*, has played an important role in increased yield during rice domestication. Only 390 bp of *GW5* can be mapped to Chromosome 5 of Nipponbare, with a larger part of the gene overlapping a 1212-bp deletion in the Nipponbare genome. Analysis of our insertion data set revealed 17 samples that contain the corresponding insertion of 1212 bp. Using historical phenotyping data we were able to confirm its association with grain weight, although the *P*-value appeared to be barely significant due to sample size (Supplemental Figure S9a).

Many studies have associated copy number variants with changes in gene expression levels and various adaptive traits. A study of the *GL7* locus (Wang et al. 2015) revealed that a 17.1-kb tandem duplication is responsible for a long-grain phenotype in selected rice varieties. We identified 111 varieties in the 3K RG data set with this causative duplication. Historical data confirmed the association with grain length (Supplemental Fig. S9b).

A study on anaerobic germination previously discovered a 20.9-kb deletion of the *AG1* locus in several varieties included in the 3K RG (Kretzschmar et al. 2015) data set. In our predicted SV data set, we found 156 samples with this deletion, 39 of which had an additional deletion of ∼100 bp within the *AG1* locus and 149 belong to the *Indica* group. In addition, we found 176 samples with a novel smaller deletion (185–467 bp) at the locus instead of the longer variant previously reported. Thus, our study confirmed the presence of known SVs in important genomic regions and expanded the set of genotypes and haplotypes with novel SVs to examine in further association studies (Supplemental Fig. S9; Supplemental Data sheet “Known SVs in Genes”).

### Utility of SV data sets for GWAS

SVs have been recognized as the causative mutations for many traits. Thus, the ability to conduct association studies with SVs should aid in gene discovery. Although SV detection is known to have a higher error rate than SNP calling, we theorize that with enough coverage, our predicted SVs can be used in genome-wide association studies (GWAS). As an example, we conducted a GWAS for a seed coat color trait using a previously published SNP data set, merged with genotype data for an insertion site (Chr 07: 6068071) at the red pericarp (*Rc*; LOC_Os07g11020) gene locus, known to be the causative mutation for this trait (Sweeney et al. 2006). Supplemental Figure S10a shows that the insertion is the most significant point in the GWAS plot at the *Rc* locus.

We also tested for SV effects associated with grain length. The major peak (Supplemental Fig. S10b) coincides with the *LONG KERNEL 3* gene, which is known to regulate grain size (Takano-Kai et al. 2009). The 350-nt-long deletion within the peak truncated the longest CDS of the gene and is likely to be the causative variation. The other peak on Chromosome 11 has an indel as the most significant variation. This region does not have annotated protein-coding genes and is more difficult to interpret.

Knowing that the presence of SVs is likely to have a significant impact on a gene function, we also identified 710 functionally characterized genes in the Q-TARO database (Yonemaru et al. 2010) that intersect with deletions (MAF > 0.005) (Supplemental Data, sheet “Q-TARO Genes”), among these, 308 are completely deleted in some rice accessions.

### Deleted genes

**Figure 4.**
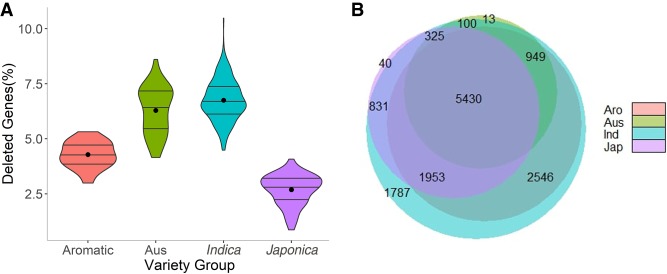
Deleted genes in variety groups. (*A*) Percentage of deleted genes in each variety group. (*B*) Number of deleted genes (frequency ≥ 5) that are unique or shared between variety groups. Note that the number of the deleted genes in *Japonica* is lower can be explained by the bias introduced by using Nipponbare genome as a reference.

We defined genes as deleted when their coding sequence is deleted over its entire length in at least one sample out of 562 high-coverage samples. Figure 4A shows the fraction of deleted genes in each sample, and Figure 4B illustrates the distributions of deleted genes among the variety groups. We used GO annotations and MAPMAN curated pathways (Thimm et al. 2004) to compare enriched biological themes of deleted temperate *Japonica* (Nipponbare) genes with the core gene set of genes that are present in all varietal groups. Results showed an enrichment of a core set genes having basal housekeeping functions and processes (e.g., developmental and catabolic processes, DNA-binding transcription factor activity, etc.). For *circum*-*Aus* and *Indica*-deleted genes, biological themes were indicative of adaptive functions/processes (e.g., response to oxidative stress, abiotic stress pathways, defense response), hinting at domestication/selection events that these variety groups underwent through their history of cultivation in diverse environments (Supplemental Data, sheet “Deleted Genes”). Deleted genes in the *circum*-*Basmati* (aromatic) group did not show any overrepresented adaptive themes, but were enriched for housekeeping functions (cell growth, carbohydrate metabolism), somewhat supporting the known cultivation history of *circum*-*Basmati* varieties in a smaller geographic region as compared to the *Indica* and *circum*-*Aus* varietal groups.

## Discussion

We present the results of one of the largest studies on structural genome variations within a crop species. We carefully assessed the performance of different methods in discovering SVs with simulated short reads. We then combined them into one pipeline and assembled a comprehensive data set of SVs produced from the 3000 rice genomes project data set.

We found ∼1.5 million SV events (clusters) longer than 9 nt that are distributed across the Nipponbare reference genome. In the manual validation, we found complex events that “confuse” the SV callers, such as short deletions in large interspersed duplications, palindromes, and terminal repeats. It is also important to note that some events may be found in large translocated regions, which could not be differentiated by the pipeline due to the limitation of detecting translocations and events contained within them. Furthermore, our deletion detection has a lower false positive rate compared to the other SV types. Although transposable elements complicate SV detection, true TE events were accurately predicted by the pipeline (see validation results).

At least 17% of the SV calls longer than 50 bp are associated with transposable elements, which contribute significantly to genomic variation in plants (Wendel et al. 2016). Based on their distribution near genes, we hypothesize that transposons play an extensive role in gene regulation, which is consistent with other studies (Naito et al. 2009; Han et al. 2013; Shen et al. 2017). Population structure, revealed by the deletion (Supplemental Fig. S4c) and CNV data sets, identified the same subgroups as genome-wide SNP analysis (Wang et al. 2018), providing additional validation of the identified SVs.

We observed a significant difference between distributions of short and long indels near TSSs. Longer deletions most frequently occurred in promoter regions, whereas short deletions were preferentially found in or near 5′ UTRs. The peak of long deletions in promoter regions at ∼360 bp upstream of the TSS is consistent with previous observations made for transposons (Han et al. 2013) and may be explained by easier accessibility of these regions for transposon insertion (Naito et al. 2009). The short deletions peak in 5′ UTRs can be explained by lower complexity of 5′ UTRs having numerous short sequence repeats. The greatest contribution to this peak was from deletions with lengths divisible by three, which is consistent with the SSR length distribution. We also detected a significant anti-correlation between the presence of SVs and TFBSs at ∼100 bp upstream of TSS, implying negative selection against deletion of important regulatory elements in these regions.

The abundance of SVs that have high sequence similarity to known transposable elements suggests that many SVs are products of TE activity. The higher number of TEs in the upstream regions of genes (Naito et al. 2009; Han et al. 2013), where promoters and regulatory motifs reside, indicates that SVs may be important agents for gene expression pleiotropy that is often observed in stress responsive genes. Studies in both human and plant genomes have found structural variants and transposable elements that are associated with aberrant expression of nearby genes (Lu et al. 2012; Wei and Cao 2016; Chiang et al. 2017). Previous studies in maize (Lu et al. 2015), cucumber (Zhang et al. 2015), soybean (McHale et al. 2012), *A. thaliana* (Debolt 2010), and 50 rice accessions (Xu et al. 2012) also associated high level of SVs in proximal locations to stress response or disease defense genes. Makarevitch et al. (2015) reported that small numbers of maize TE families may contribute to abiotic stress responses by providing stress responsive enhancer-like functions to nearby genes. They also reported that specific insertions of TEs near genes are often polymorphic within a species, in agreement with our observations across the 3K RG.

Although third-generation sequencing technologies can assemble high-quality rice genomes and assess structural variation through comparative genomics, it is unlikely that for the foreseeable future they will be applied to a large set of varieties within a species. Hence, bioinformatic analysis of short reads is currently the most practical way to assess the diversity of structural variation within a species as performed in this study. Future studies may use different reference genomes from other variety groups, and inclusion of new samples resequenced at higher depths would allow better profiling of longer insertions. Our SV data set will enable rice geneticists to explore variability that is normally missing in SNP-based genome-wide association studies. Moreover, the variability described in this analysis can be used as a hypothesis generator to identify genetic causes of different important traits through future functional studies.

## Methods

### Evaluation of SV callers

We benchmarked a set of SV callers to identify a subset to integrate into a discovery pipeline. The benchmarking pipeline was designed so that the performance of SV callers could be evaluated with respect to variant types—deletion (DEL), insertion (INS), inversion (INV), tandem duplication (DUP), and translocation—and variant sizes, binned according to lengths: A (50–150 bp), B (151–500 bp), C (500–5000 bp), D (5–50 kb), E (50–250 kb), and F (0.25–1 Mb). Test data were designed to replicate the 14× average coverage of the 3K RG data set with 1000 introduced variations per SV type for the first four bins, and 200 and 100 variations for bins E and F, respectively. Genomic sequences with DEL, DUP, and INV variants were created by SVSIM (https://github.com/mfranberg/svsim) using the Nipponbare RefSeq (IRGSP 1.0), and sequencing reads were simulated by WGSIM (https://github.com/lh3/wgsim) with 83-bp read lengths, 500-bp insert sizes (SD = 50), and 0.02 error rates. It is worth clarifying that all DUP events simulated by SVSIM were tandem duplications (TDs). For insertion types, simulated paired-end reads of the Nipponbare RefSeq were aligned to another reference genome with randomly deleted regions. For translocation types, random regions in the Nipponbare RefSeq were deleted and inserted into regions either in the same or another chromosome. The Burrows–Wheeler Aligner's (BWA) (Li and Durbin 2010) paired-end module was used in mapping reads to a reference.

A prediction of an SV caller was considered correct if it passed 90% minimum reciprocal overlap (RO) and its breakpoint error (*e*), defined as the sum of distances between the breakpoint starts and ends of the predicted SV(*p*) and the simulated event (*S*), was less than 10 bp (allowing for microhomologies around breakpoint sites) (Schröder et al. 2014) or <10% of *length(p)+length(S)* (requiring less error for events with size <50 bp). However, these conditions were too strict to detect duplications that have poor breakpoint resolutions. In Supplemental Figure S11, the sensitivity of the callers on different ROs suggested a 70% threshold and no constraint for *e* to evaluate fairly duplication breakpoints.

### Discovery pipeline

Based on benchmarking results, Pindel (Ye et al. 2009) was selected as the main variant caller for the pipeline since it consistently called more precise predictions across almost all bins of deletions, tandem duplications, and inversions even though it had relatively lower sensitivity for the largest events. DELLY (Rausch et al. 2012), GROM (Smith et al. 2017a), and LUMPY (Layer et al. 2014) were added to improve sensitivity and support of predictions especially for larger variants. For insertions, both MetaSV (Mohiyuddin et al. 2015) and MindTheGap (Rizk et al. 2014) were chosen to complement each other for better sensitivity for short and long insertions in the 562 highest-coverage samples since assembly-based algorithms require high coverages for accurate prediction. Interspersed duplications can only be detected using read depth signals; therefore, we analyzed copy number variation (CNV) predictions from NGSEP (Duitama et al. 2014) as a separate data set.

Given the calls predicted in each sample, merging results from all callers required a minimum reciprocal overlap (RO) to classify whether or not calls were similar. A common merging strategy is to require each variant prediction to be supported by at least two callers; however, this may increase false negatives when some selected callers perform poorly for some size ranges. To address this, our pipeline retained

1. all Pindel results with QUAL = PASS, regardless of other callers’ support; for inversion, all Lumpy calls; and
2. results from other callers, not supported by Pindel, when supported by at least two callers, using the following criteria: If merging of callers is between Lumpy and another caller, 70% RO was required; otherwise, 90% RO.

These criteria were applied to all SV types except insertions that require a different clustering approach.

Supplemental Figure S12 shows the breakpoint accuracy of the callers based on sensitivity improvement using either 70% or 90% RO. The 90% threshold for reciprocal overlap was reduced to 70% to consider inaccuracy of duplication breakpoints from Lumpy. To merge insertion calls from MetaSV and MindTheGap, the pipeline clustered all INS sites that were within 10 bp of one another and included all unique insertions assembled by the callers.

To create a map of variant sites across all samples, we clustered variant calls across different samples and pooled them as follows. All events that overlapped by at least 1 bp were initially grouped together. For each group, a graph was built with SVs as nodes with edges connecting SVs that have at least 90% reciprocal overlap, or at least 70% RO with a *breakpoint error* of at most 10 bp. The latter condition allows for grouping of small events from different samples that have <90% RO but very small boundary differences. Each group was then split into connected components of the graph. For each connected component, we computed a distance matrix using the absolute value of the distances between breakpoints of two variants divided by their total lengths. Then, hierarchical clustering by complete linkage was performed using the distance matrix with a cutoff of 0.1 for height, yielding the final clusters. We consider each cluster to represent a single ancestral event inherited by a subset of our sample. This stage is done for each variant type except insertions, which were clustered by grouping events that are at most 10 bp apart.

The pipeline described above uses SV callers selected for their performance on detecting insertions, deletions, inversions, and tandem duplications. However, the use of read depth signals allows for the discovery of larger copy number variants, including interspersed duplications and those variants located in complex genomic regions that RP and SR methods find difficult to detect (Medvedev et al. 2009; Zhao et al. 2013). NGSEP, one of the callers using RD signals, was used to compile a CNV data set (Supplemental Table S3).

### Validation

With the published “N 22::IRGC 19379-1” hereafter referred to as N 22 (NCBI Assembly ASM195236v1) (Stein et al. 2018) and Nipponbare reference IRGSP 1.0 (Kawahara et al. 2013), we validated random SVs predicted in CX368, an N 22 accession in the 3K RG data set. Random SVs were selected and manually inspected in a dot plot alignment between N 22 and Nipponbare generated using Gepard (Krumsiek et al. 2007). Events found in long deleted regions were further analyzed using NCBI BLAST to determine if they occur in translocated regions in the Nipponbare reference. False positive rate was computed per SV type depending on the number of predicted calls that were inconsistent with the dot plots. Some false positive calls may also be private to CX368 (a different N 22 sample) and not to the N 22 reference genome. Because we mainly expect to discover tandem duplications using the pipeline, interspersed duplications were given lower weights of being true positives. For computing false negative rates, we focused on deletions and identified 20 events between N 22 and Nipponbare using the dot plots in randomly selected locations and validated if they were predicted in CX368.

The scripts used for the structural variant discovery pipeline are available at https://github.com/rrfuentes/SV_Discovery as well as in Supplemental Code.

### Identification and analysis of transcription factor binding sites

We extracted regulatory regions [TSS-500, TSS + 500] for all “high confidence” rice genes defined by Tatarinova et al. (2016). The distribution of transcription factor binding sites (TFBSs) in these regulatory regions were analyzed with the MATCH algorithm (Kel et al. 2003) using the TRANSFAC database (Wingender et al. 1996) comprised of 764 plant position weight matrices. For each genomic position, we calculated the fraction of genes that have a TBFS in this position and computed a probability that each position *x* is covered by a putative regulatory element, *P*_*x*_(*TFBS*). Then for each position in this region, we calculated the fraction of all genes that have a structural variant and TBFS covering the same position, resulting in the probability *P*_*x*_(*TFBS ∩ SV*). The conditional probability was calculated as follows:

$$P_{x}(SV|TFBS)=\frac{P_{x}(TBFS\cap SV)}{P_{x}(TFBS)}.$$

### Sequence complexity

To rule out the influence of sequence complexity on the efficiency of read mapping and variant calling, we calculated sequence complexity profiles around transcription start sites. For every sequence around a transcription start site [TSS − 1000, TSS + 1000], we calculated the Linguistic Complexity (CL)

$$CL=\left(\sum_{i=1}^{N}V_{i}\right)/\left(\sum_{i=1}^{N}V_{maxi}\right),$$

where *N* is the window size (10 bp in our case), *V*_*i*_ is the number of words of size *i* in the window, and *V*_*maxi*_ is the maximum possible number of words of length *i*. For a window of size *N*, and alphabet size *K*, this number is calculated according to the following formula: *V*_*maxi*_ = min (*K*^*i*^, *N* − *i* + 1) (Orlov and Potapov 2004). CL is the ratio of the observed number of different words of size 1, …, *N* in a given window divided by the sum of maximum possible number of different words for a fixed window length. After calculating the complexity profile for every sequence, we averaged across all rice promoters.

### Deleted genes

For each sample, all non-TE genes that were completely deleted were identified and their sequences retrieved. These sequences were compared against all insertion sequences to remove all of those that may have been translocated or have similar copies in other parts of the genome. Using the variety group assignment from Wang et al. (2018), we computed the average number of genes deleted per variety group. This analysis focused on genes present in the Nipponbare genome.

### Gene enrichment analysis

To identify overrepresented (enriched) biological themes of genes covered by large deletions, we compared the list of these subset genes against the “population” of genes in the genome that is annotated within a given system of classifying genes (e.g., “Biological Process” in Gene Ontology). “Hits” refers to genes in the population falling within the gene category in question. As an example, “Population hits” for the GO annotation Biological Process “cellular stress response to acidic pH” refers to the number of genes falling within the category “cellular stress response to acidic pH” out of all genes in the population annotated with a Biological Process. Given the number of genes in the subset SV gene list that fall within a specific category (the “List hits”), the count of genes in the list (the “List total”) and the corresponding “Population Hits” and “Population Total,” the probability of seeing the number of “List Hits” in the “List Total” given the frequency of “Population Hits” in the “Population Total” is calculated with the Fisher's exact test and reported for overrepresentation analysis.

### Genome-wide association studies

We conducted GWAS on the seed coat color and grain length phenotypes, recorded by the T.T. Chang Genetic Resources Center for the International Rice Genebank Collection information system at IRRI and retrieved from SNP-Seek, on a 365 high-sequence coverage subset of the 3K RG where the phenotype was measured: (“white”) 291 samples; (“red”) 74 samples. To construct the genotype file, we first merged two data sets (GATK SNP and small indels and the INS data set). Then, we merged this single-variant data set with a filtered and LD-pruned SNP data set (MAF > 0.015, max miss = 0.2, *r*^2^ ≤ 0.8 within 2 kb, total 889,903 SNPs).

We used linear mixed model association analysis implemented in GEMMA (Zhou and Stephens 2012), using a kinship matrix and the first five principal components for relatedness and population structure correction. The kinship matrix was computed by the GEMMA -gk command with default parameters. The PCA was computed using PLINK 1.9 (Purcell et al. 2007). We plotted Manhattan and QQ plots using the R package “qqman” (Turner 2014) with in-house modifications for graphics.

## Data access

All SVs identified in this study are available at the SNP-Seek portal (http://snp-seek.irri.org) in the download section.

## Supplementary Material

Supplemental Material
