# Supplementary material for: Structural variants in 3000 rice genomes
Source: Genome Res. 2019 May;29(5):870–80. doi: 10.1101/gr.241240.118 (PMC6499320; doi:10.1101/gr.241240.118)
Supplement: Supplemental Material [file supp_29_5_870__index.html]

Structural variants in 3000 rice genomes — Structural variants in 3000 rice genomes — Supplemental Material 

# Structural variants in 3000 rice genomes

## Supplemental Material

- Supplemental\_Data.xlsx
- Supplemental\_Code.zip
- Supplemental\_Material.pdf
