# Supplementary material for: Structural variants in 3000 rice genomes
Source: Genome Res. 2019 May;29(5):870–80. doi: 10.1101/gr.241240.118 (PMC6499320; doi:10.1101/gr.241240.118)
Supplement: Supplemental Material [file supp_gr.241240.118_Supplemental_Material.pdf]

SUPPLEMENTAL INFORMATION

Supplemental Figures

Figure S1. Benchmark of structural variants callers using simulated events. **2**
Figure S2. Breakpoint differences between members of each structural variation cluster. **3**
Figure S3. Comparison of SVs detected by Wang et al 2018 with our SV dataset. **4**
Figure S4. Principal components analysis for the DEL dataset using all (a, b) and 560 high-coverage samples (c, d). **5**
Figure S5. Distribution of short deletions around transcription start sites (TSSs). **6**
Figure S6. Average sequence complexity around the vicinity of transcription start sites. **8**
Figure S7. SNP versus SV densities in 100kb sliding windows across the Nipponbare RefSeq. **9**
Figure S8. SNP and SV distribution across the genome and colocalization of SV peaks and gene classes. **10**
Figure S9. Known structural variants verified using the dataset. **11**
Figure S10. Genome-wide association studies. **12**
Figure S11. Sensitivity of callers on duplication prediction based on different limits for reciprocal overlap. **13**
Figure S12. Ratio of caller sensitivity on 70% RO over 90% RO. **14**
Figure S13. Impact on the rice genome of structural variants predicted by NGSEP. **15**
Figure S14. Structure and enrichment analysis of CNVs. **16**
Figure S15. Distribution of short deletions around TSS. **17**
Figure S16. Distribution of the number of deletions in the vicinities of start and end of transcription and translation. **18**
Figure S17. Dot plots for selected variants validated as true positives. **19**
Figure S18. Assessment of copy number variation prediction. **20**
Figure S19. Comparison of deletion and insertion sequences to known or potentially active TEs. **21**

Supplemental Tables

Table S1. Transposable and repeat elements (size >50bp) among the structural variants. **22**
Table S2. Strategies and SV types supported by each caller. **23**
Table S3. Summary of structural variants (SV) identified by NGSEP on the complete dataset combining read depth (RD), read pair (RP) and split read (SR) approaches. **24**
Table S4. Manually validated SVs using N22 (CX368) reference genome. **25**

Supplemental Methods **26**

Figure S1. Benchmark of structural variants callers using simulated events. (a) F1-score (harmonic average of the precision and recall) of each caller is computed per variant type and size bin. Sizes are binned according to lengths: A (50-150 bp), B (151-500 bp), C (500-5000 bp), D (5-50 kb), E (50-250 kb) and F (0.25-1Mb). (b) Sensitivity of GATK-UG and the SV discovery pipeline according to the sizes of variants**.** GATK-UG is sensitive only for short insertions and deletions and must be complemented by other variant callers, such as our pipeline built for structural variants. (c) Percentage of GATK-UG within SV-pipeline deletions predicted in random samples, stratified by deletion size.

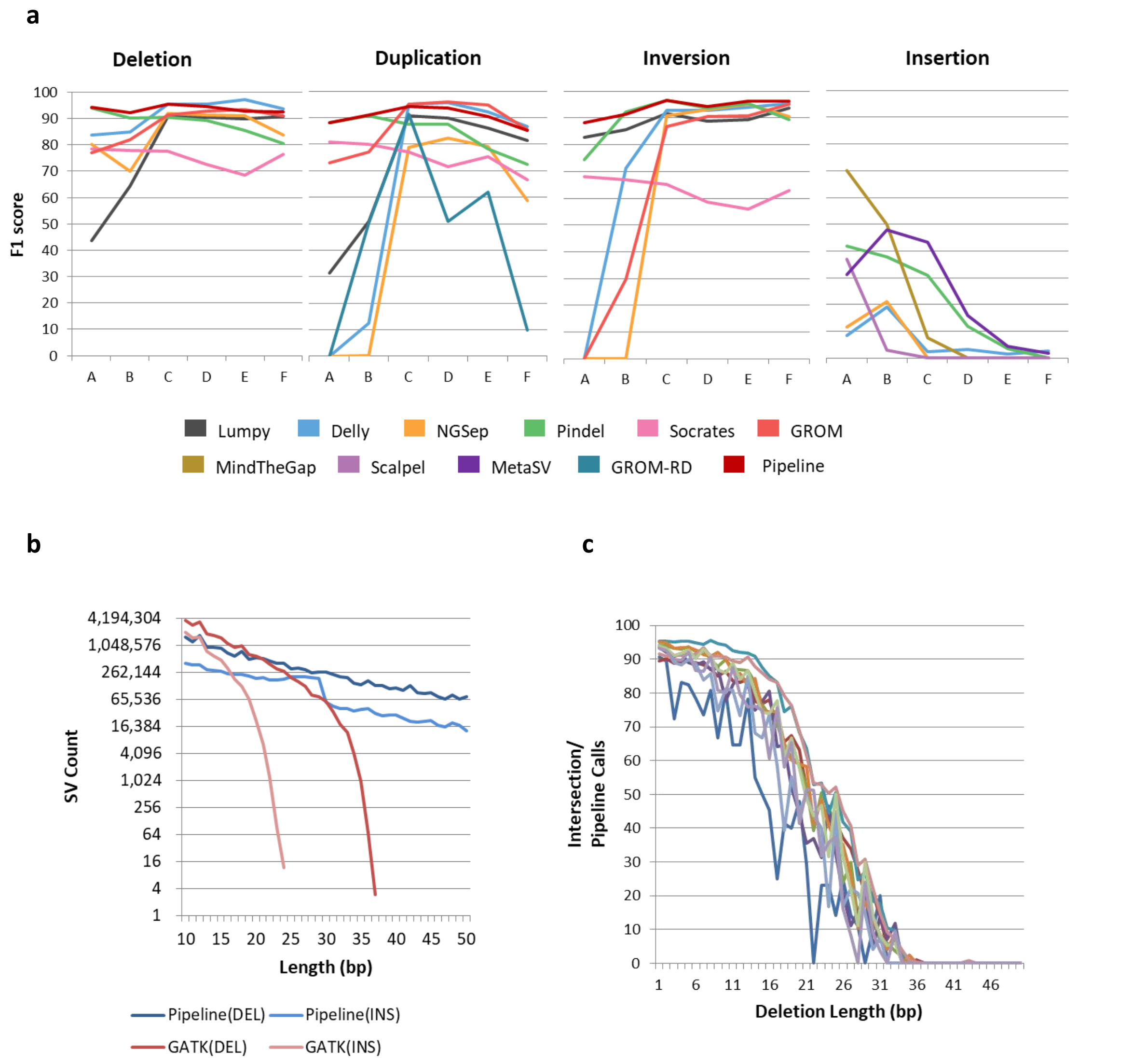

Figure S2. **Breakpoint differences between members of each structural variation cluster.** After implementing hierarchical clustering, groupings were refined and the computed mean distance is 2.2%.

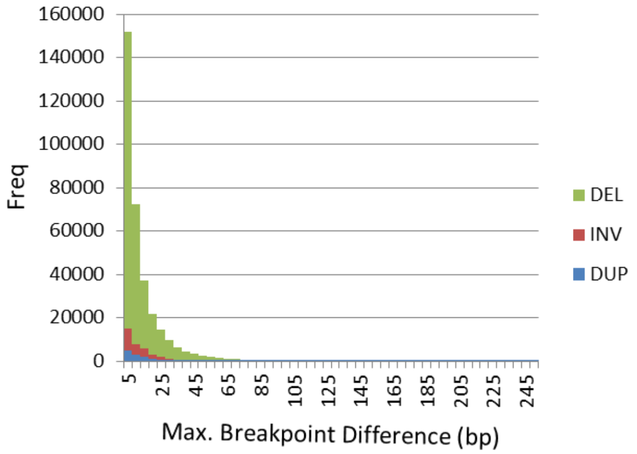

Figure S3. Comparison of SVs detected by Wang et al 2018 with our SV dataset. For a fair comparison we applied the same filtering as in Wang et al: size between 100 bp and 500 kb; frequencies between 6 samples and 80% of samples; only SVs from the 453 subset samples were used. Translocations were excluded from this comparison due to the poor performance of the callers in detecting this specific SV type. The comparison of our clusters with Wang et al clusters is not one-to-one due to differences in the clustering method; thus, the circle intersections list both the number of Wang et al clusters that have >50% reciprocal overlap with our clusters (blue), and the number of our clusters (orange). Large differences in cluster number are due to the different clustering approaches. Our full dataset, including all samples and variants of size below 100 bp, is represented by the broken circle.

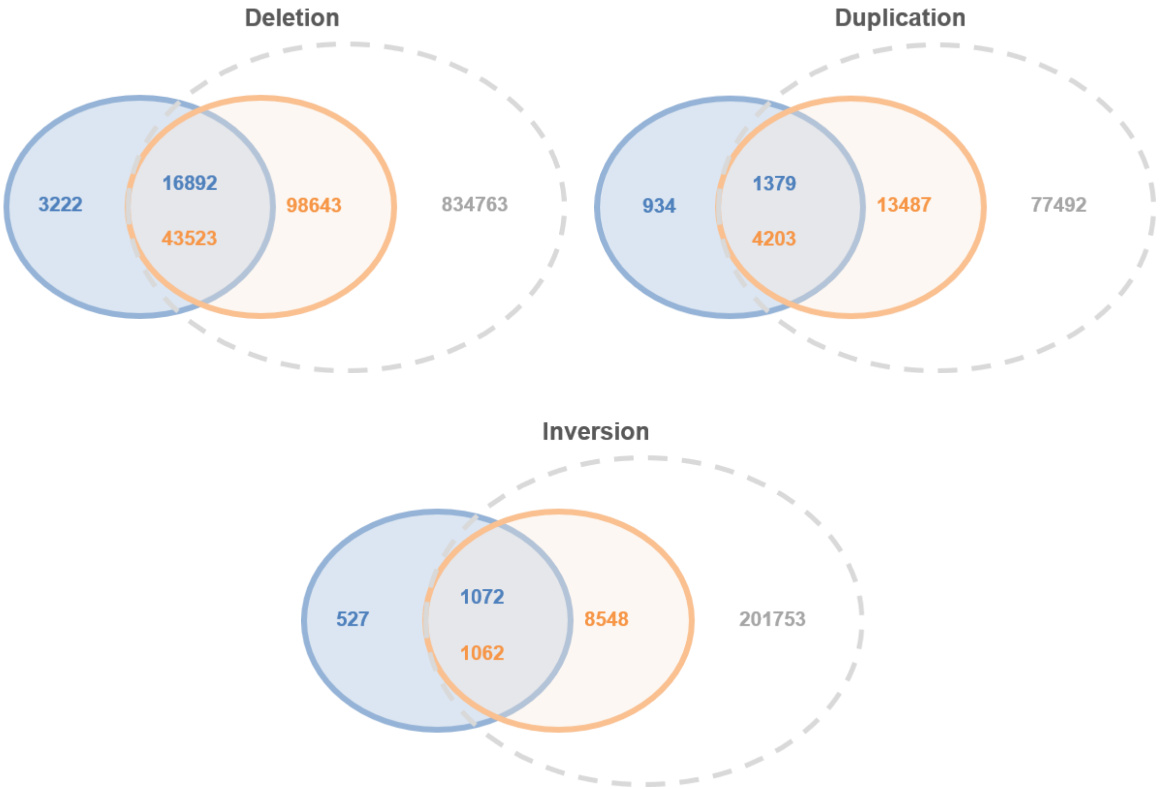

Figure S4. Principal components analysis for the DEL dataset using all (a, b) and 560 high-coverage samples (c, d). The color indicates clusters from Wang *et al.* defined by SNP data.

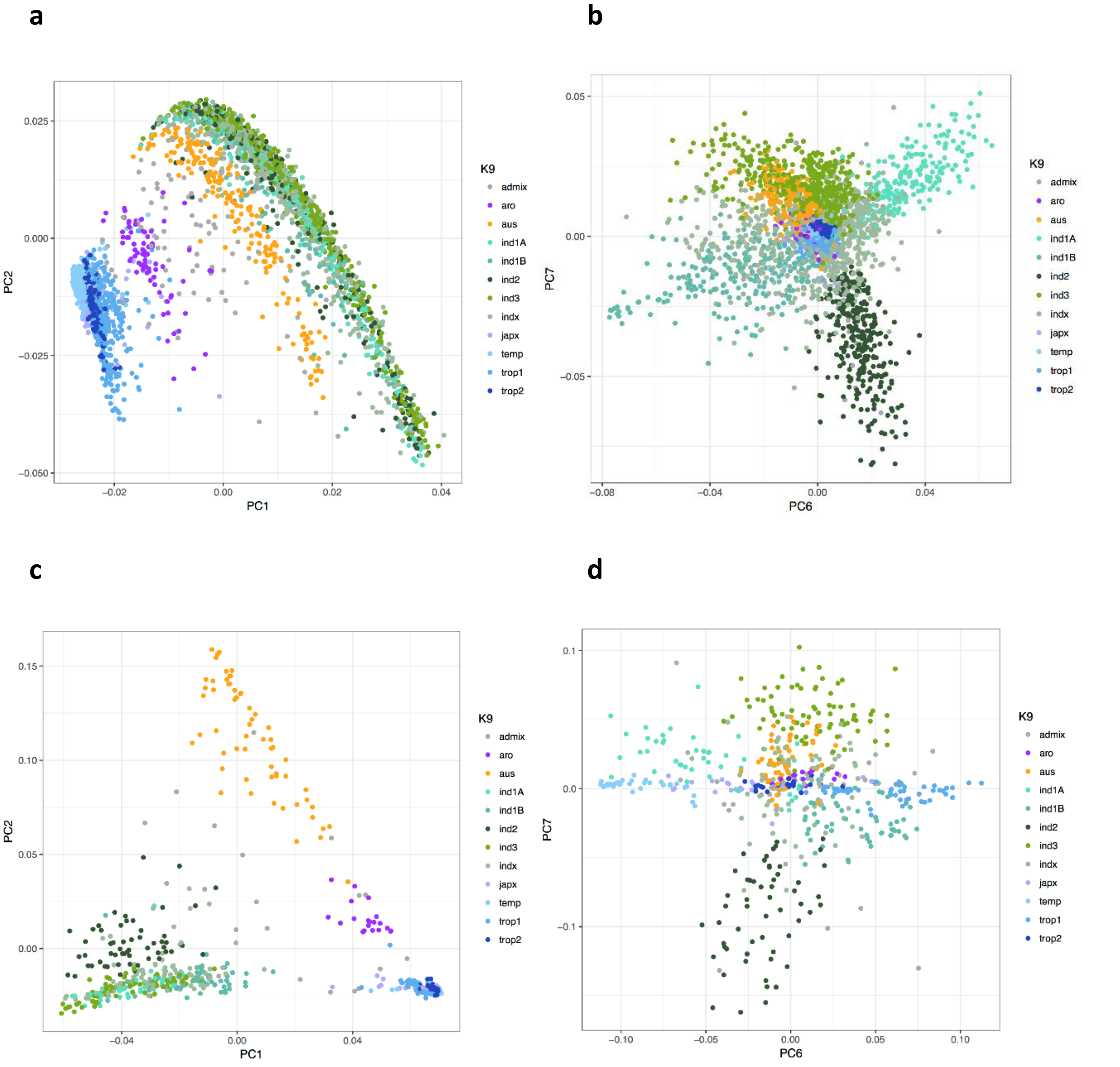

Figure S5. Distribution of short deletions around transcription start sites (TSSs). Deletions with sizes in multiples of 3 nucleotides contribute largely to the peak in the 5’ UTRs, and they have a 4-fold lower distribution on both sides of these regions.

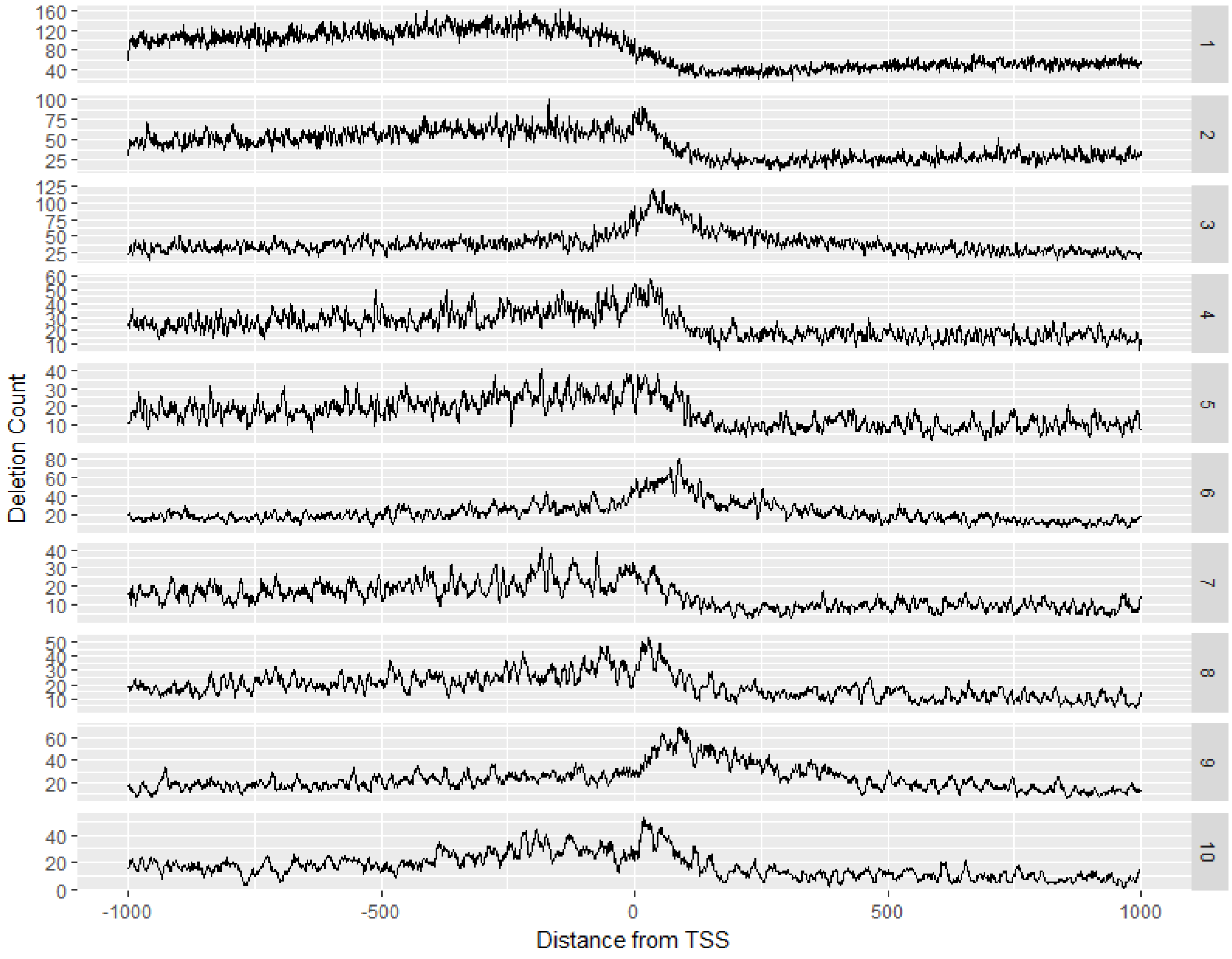

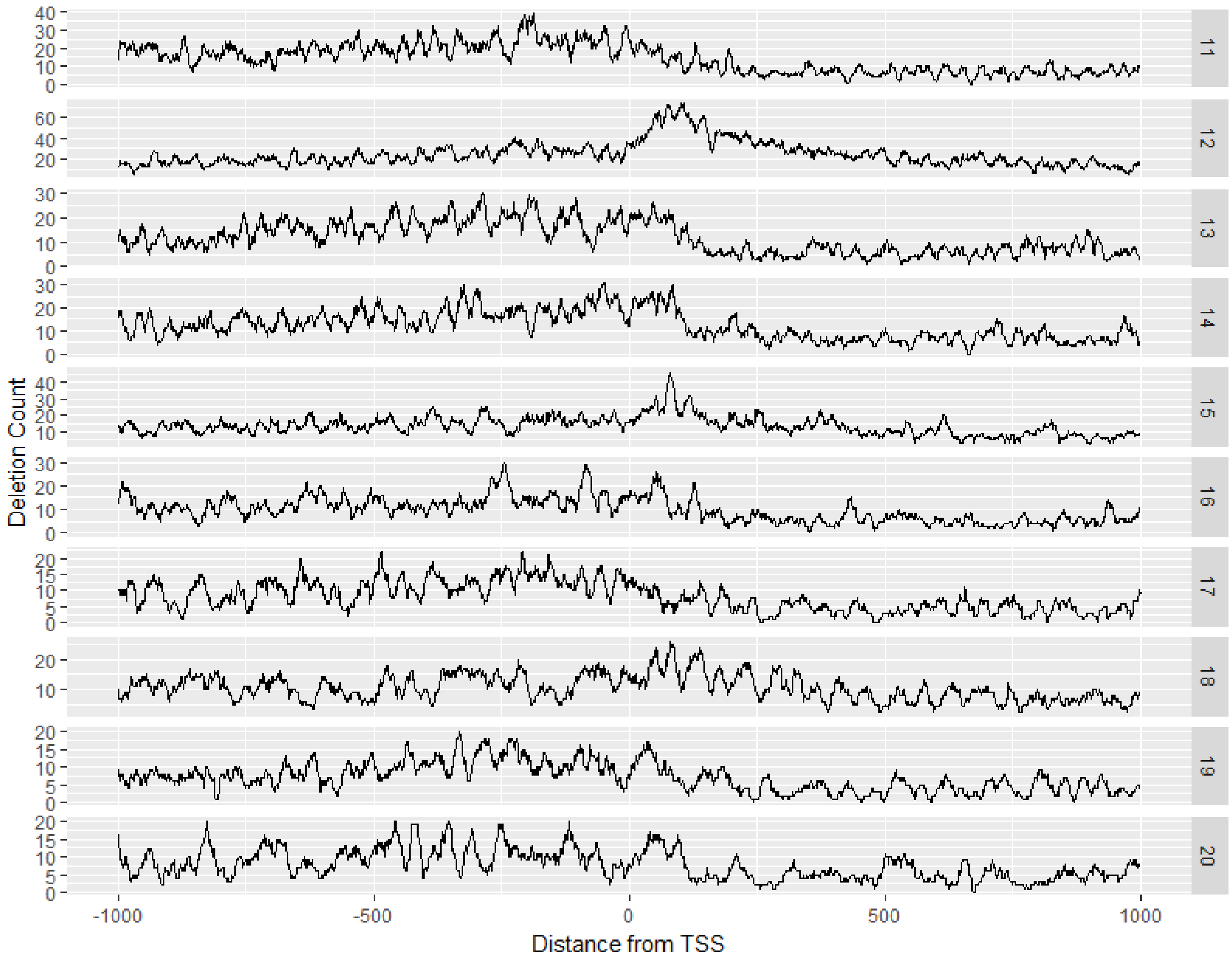
Deletion Count
Distance from TSS
-1000
-500
0
500
1000
11
12
13
14
15
16
17
18
19
20

Figure S6. Average sequence complexity around the vicinity of transcription start sites.

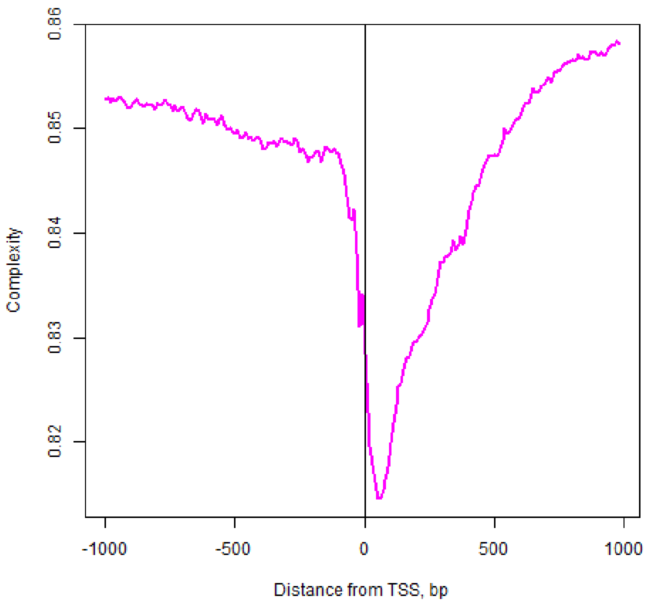

Figure S7. SNP versus SV densities in 100kb sliding windows across the Nipponbare RefSeq. SNP data were taken from SNP-Seek database (Mansueto *et. al.* 2017).

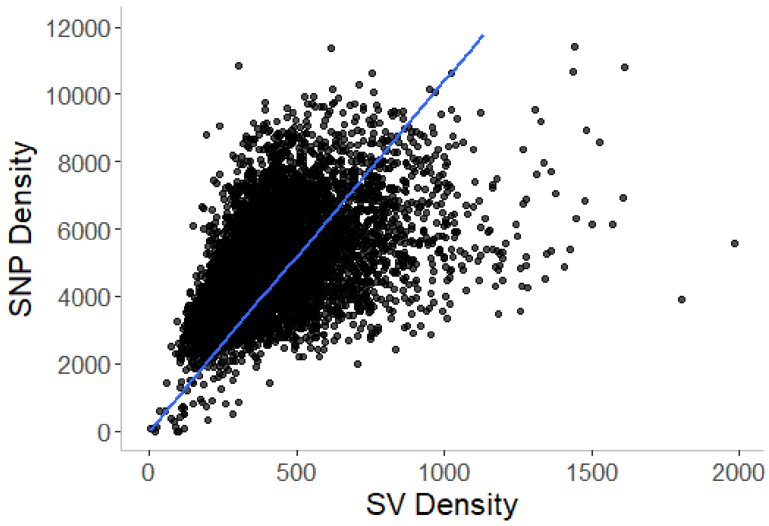

Figure S8. SNP and SV distribution across the genome and colocalization of SV peaks and gene classes.

The red and black lines shows the correlated distribution of SNPs and SVs, respectively, across the chromosomes. Dots indicate regions with higher numbers of structural variants that contain genes associated with stress and other responses.

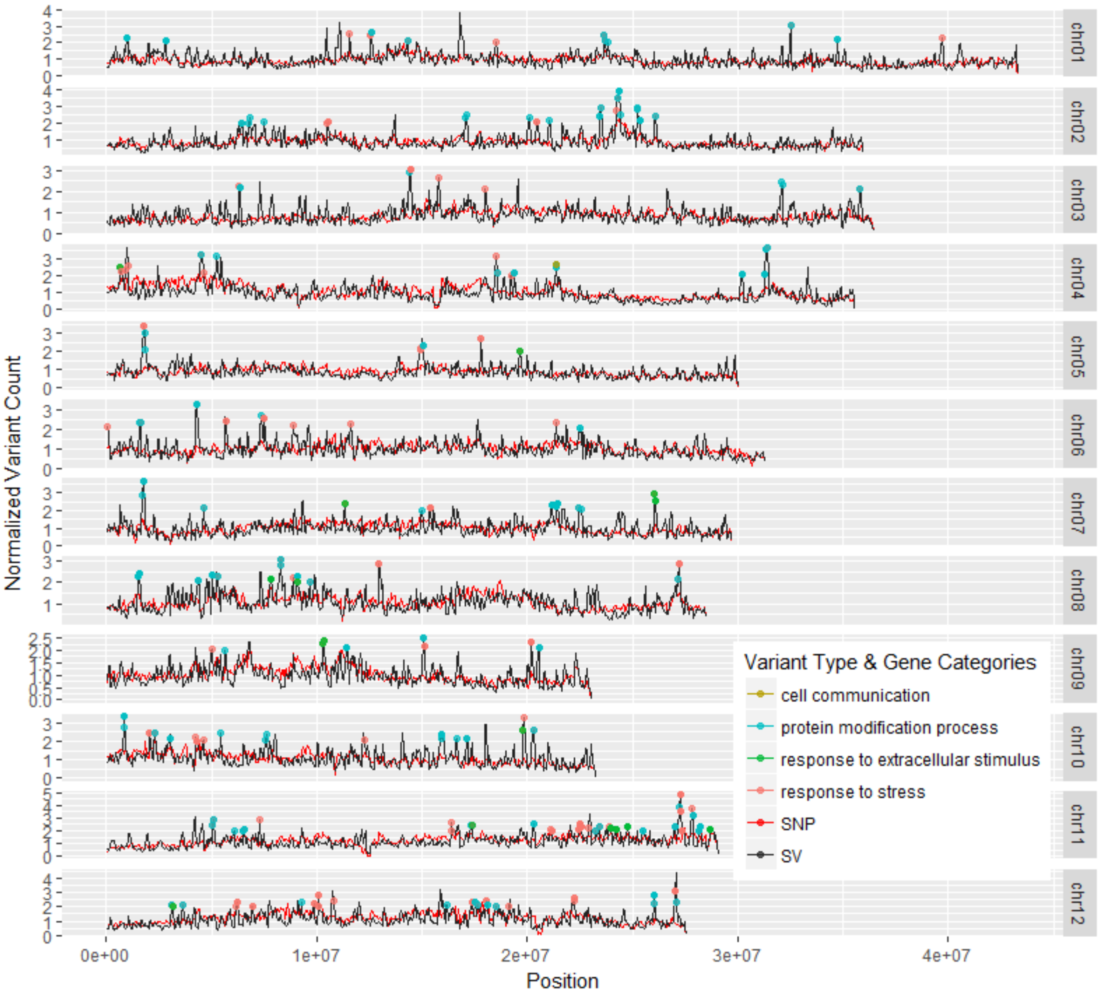

Figure S9. Known structural variants verified using the dataset. a) *GW5* gene is partially deleted from the Nipponbare RefSeq (green) and effect of the observed 1212 bp insertion on grain weight, p-value=0.04. **b**) tandem duplication near *GL7 (LOC_Os06g41200)* gene and effect of the event on grain length, p-value = 1.0e-10.

a

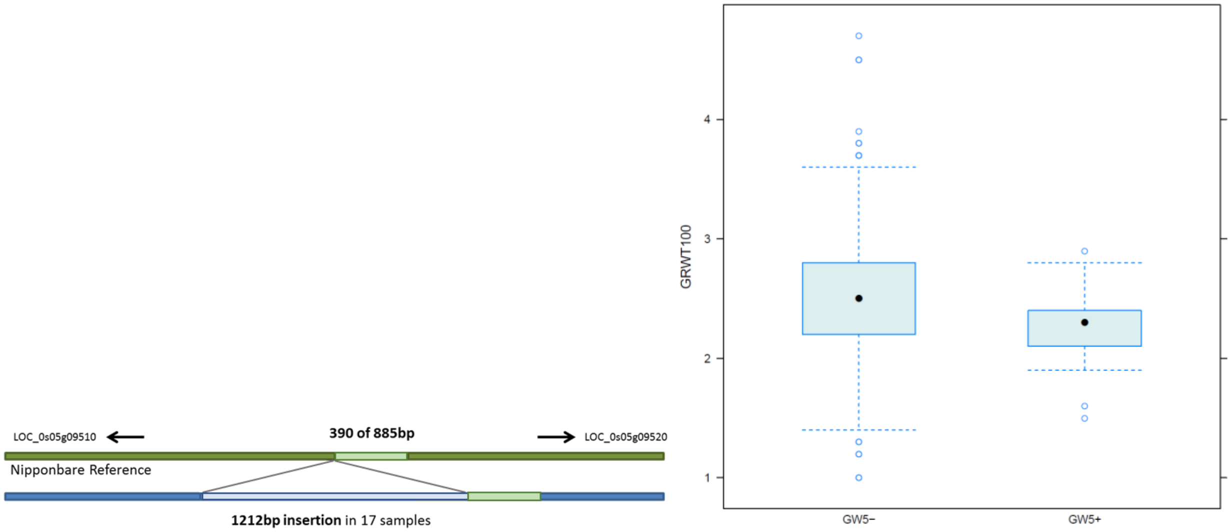

b

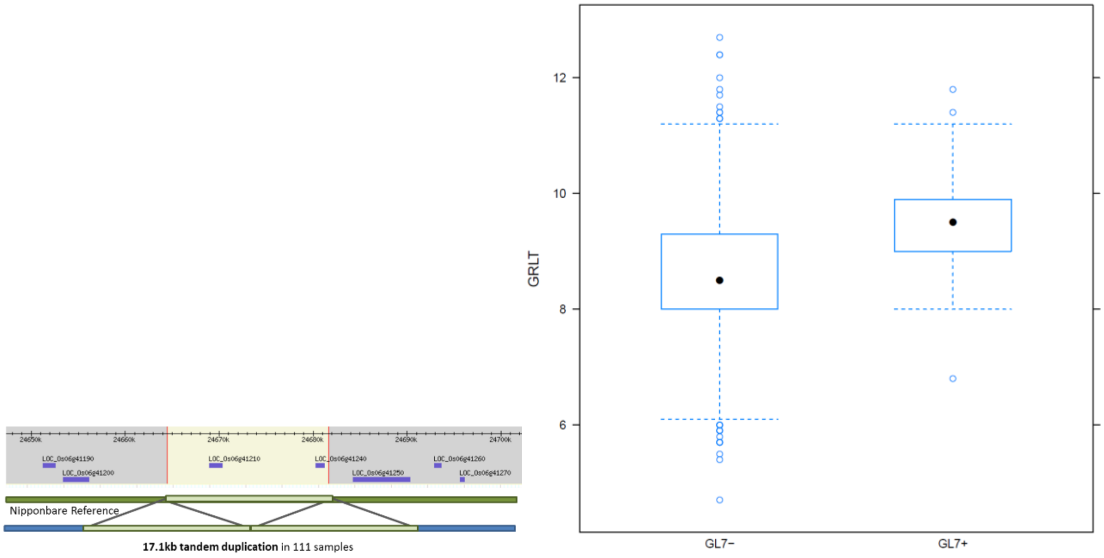

Figure S10. Genome-wide association studies. Manhattan and QQ plots for the **a**) *Rc* (red pericarp) trait and **b**) grain length trait.

a

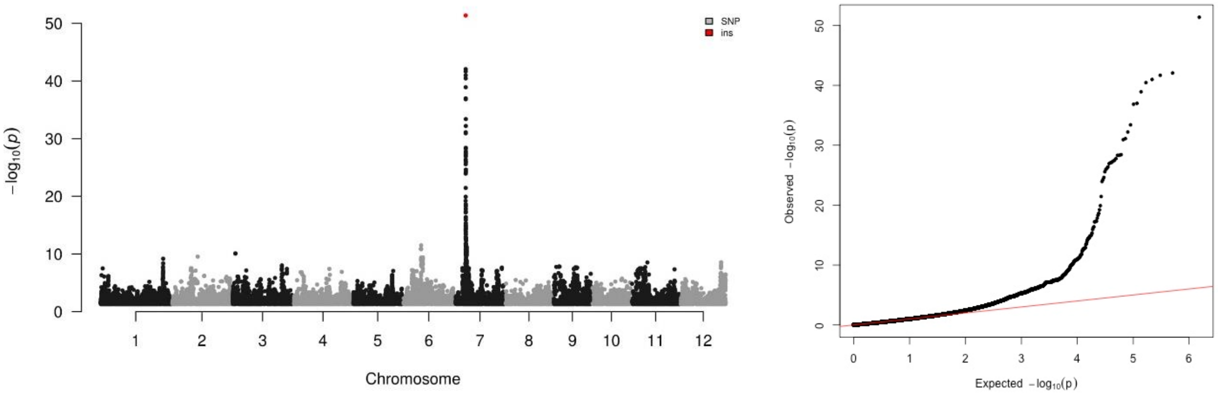

b

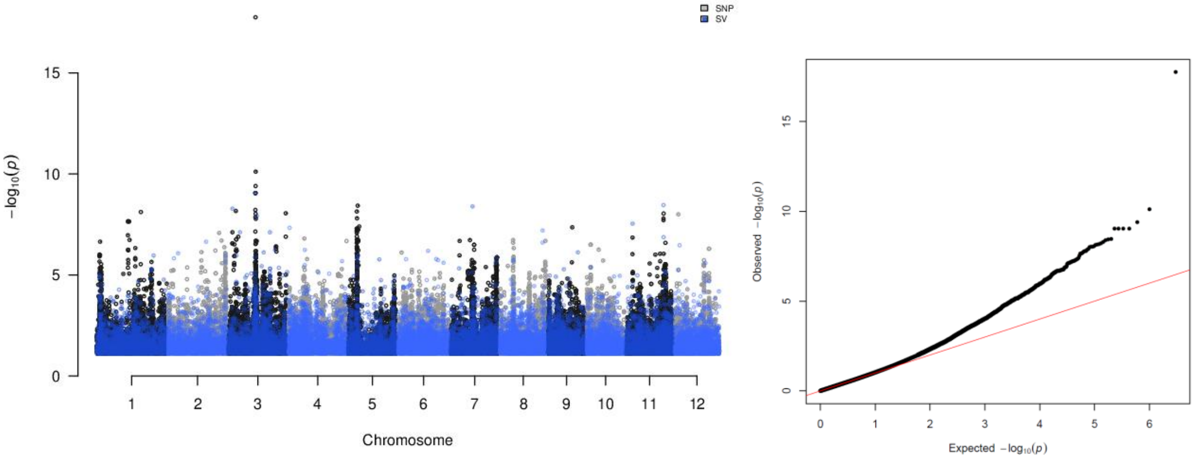

Figure S11. **Sensitivity of callers on duplication prediction based on different limits for reciprocal overlap**. The profiles of sensitivity show robustness of callers to the reciprocal overlap parameters.

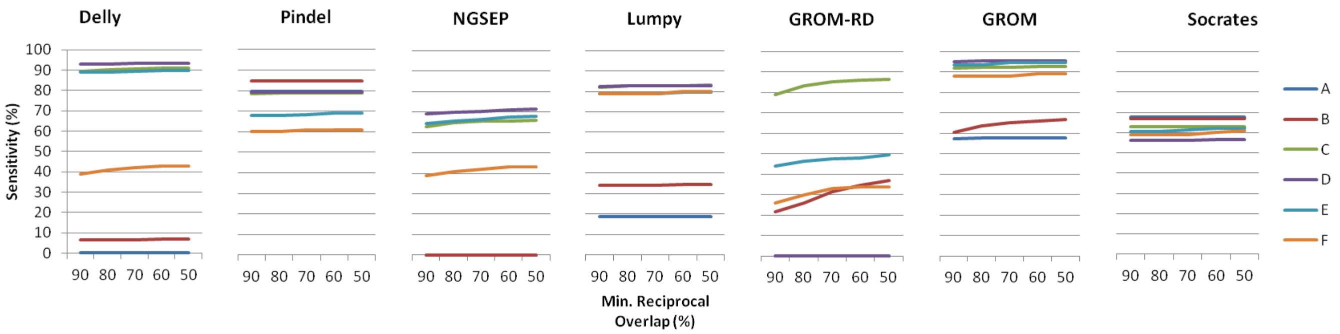

Figure S12. **Ratio of caller sensitivity on 70% RO over 90% RO.** A ratio of 1.0 means that the sensitivity did not change after relaxing the reciprocal overlap (RO) threshold. A near horizontal line shows reliability of breakpoint resolution across different size bins. Both Socrates (Schröder et al. 2014) and Pindel (Ye et al. 2009) consistently predicted precise breakpoints, thus reducing RO to 70% will barely improve their sensitivity.

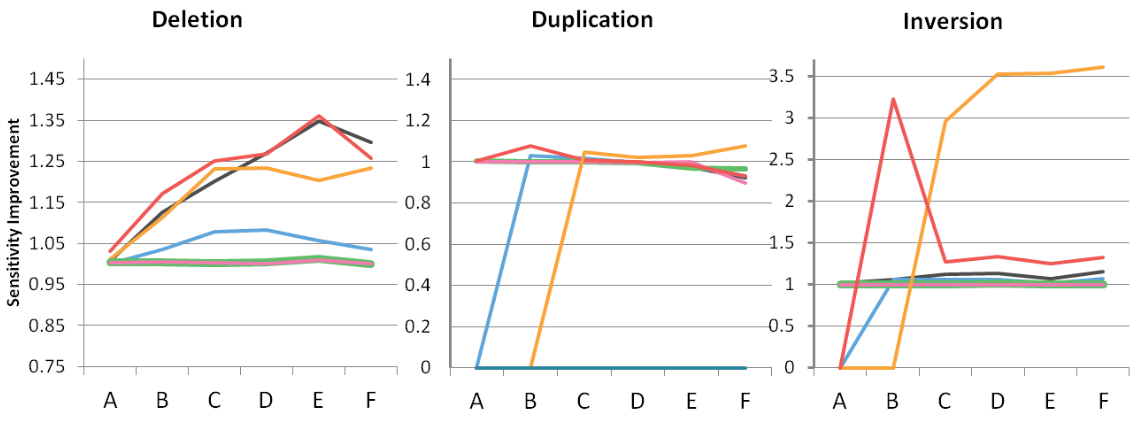

Figure S13. Impact on the rice genome of structural variants predicted by NGSEP. a) Length distribution of raw structural variants called by NGSEP. b) Distribution of the amount of reference genome covered by CNV structural variants across the 3K RG samples.

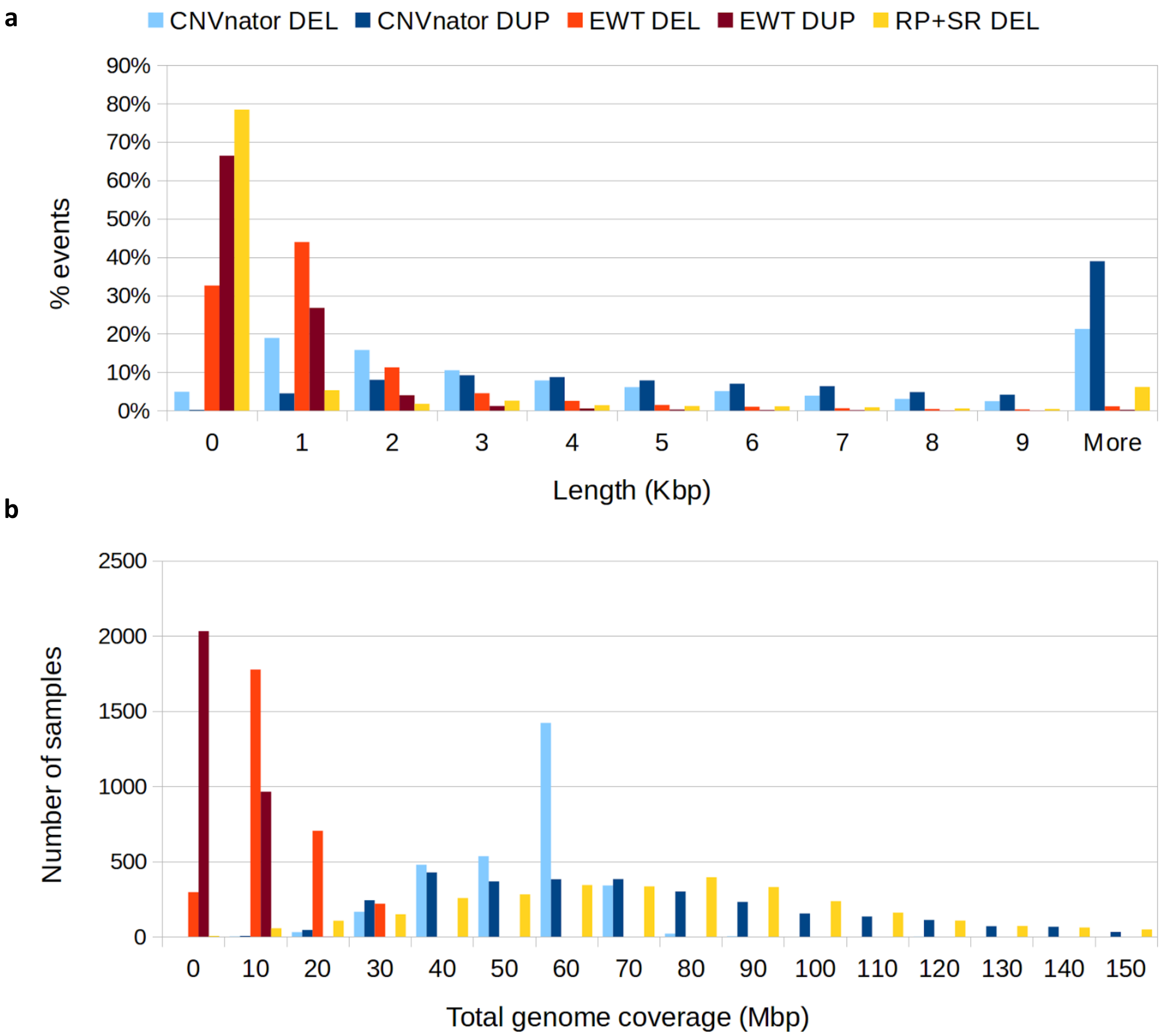

Figure S14. Structure and enrichment analysis of CNVs. a) Structure analysis using 2,839 CNV genotyped in at least 2,000 of the 3,023 samples, in non-repetitive regions of the genome and having the major allele in at most 80% of the samples. Enriched biological process (**b**) molecular function (**c**) gene ontology terms in genes covered by CNVs.

a

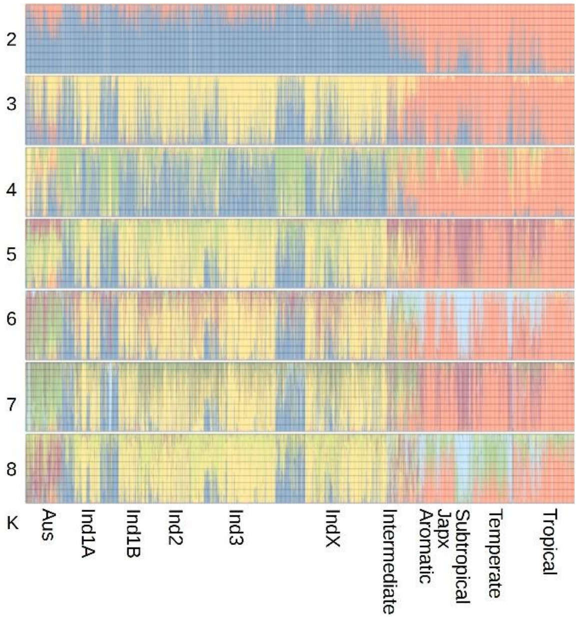

b

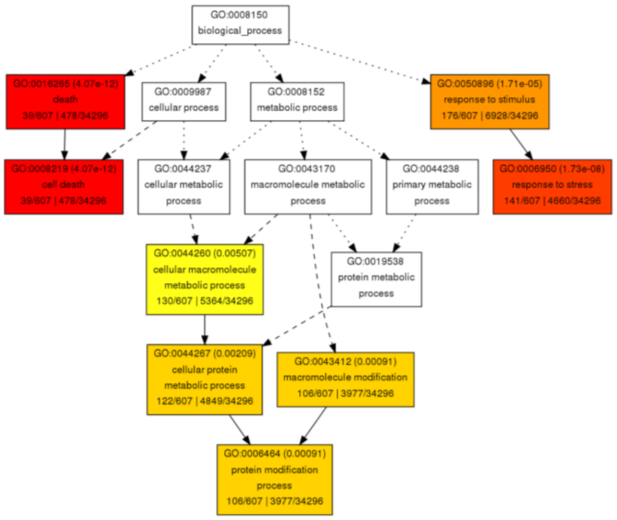

c

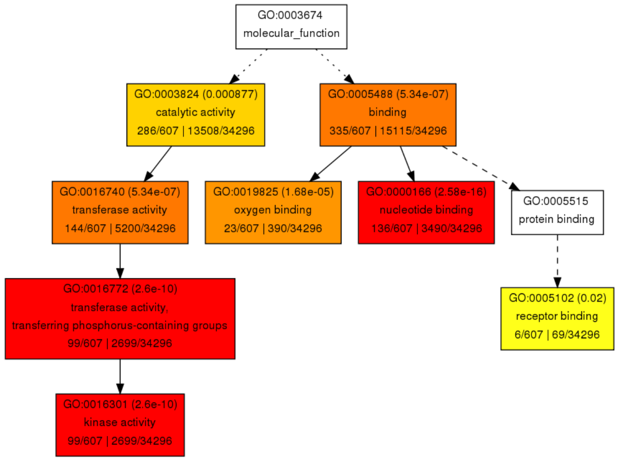

Figure S15. Distribution of short deletions around TSS. The plot shows a peak of deletions with size in multiples of 3 bp downstream of TSS.

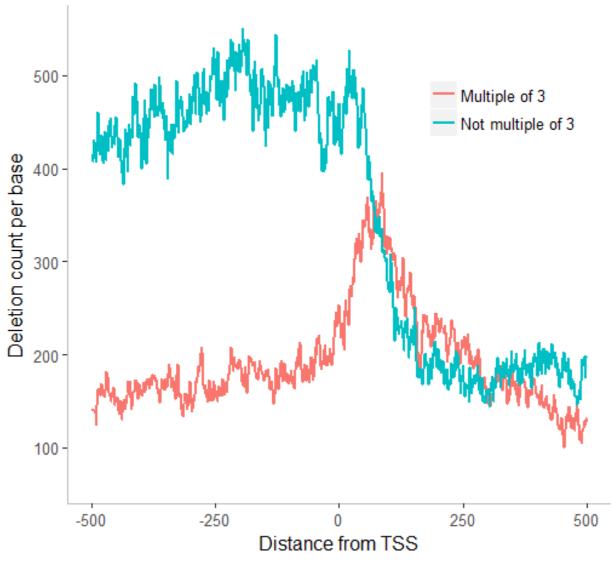

Figure S16. Distribution of the number of deletions in the vicinities of start and end of transcription and translation.

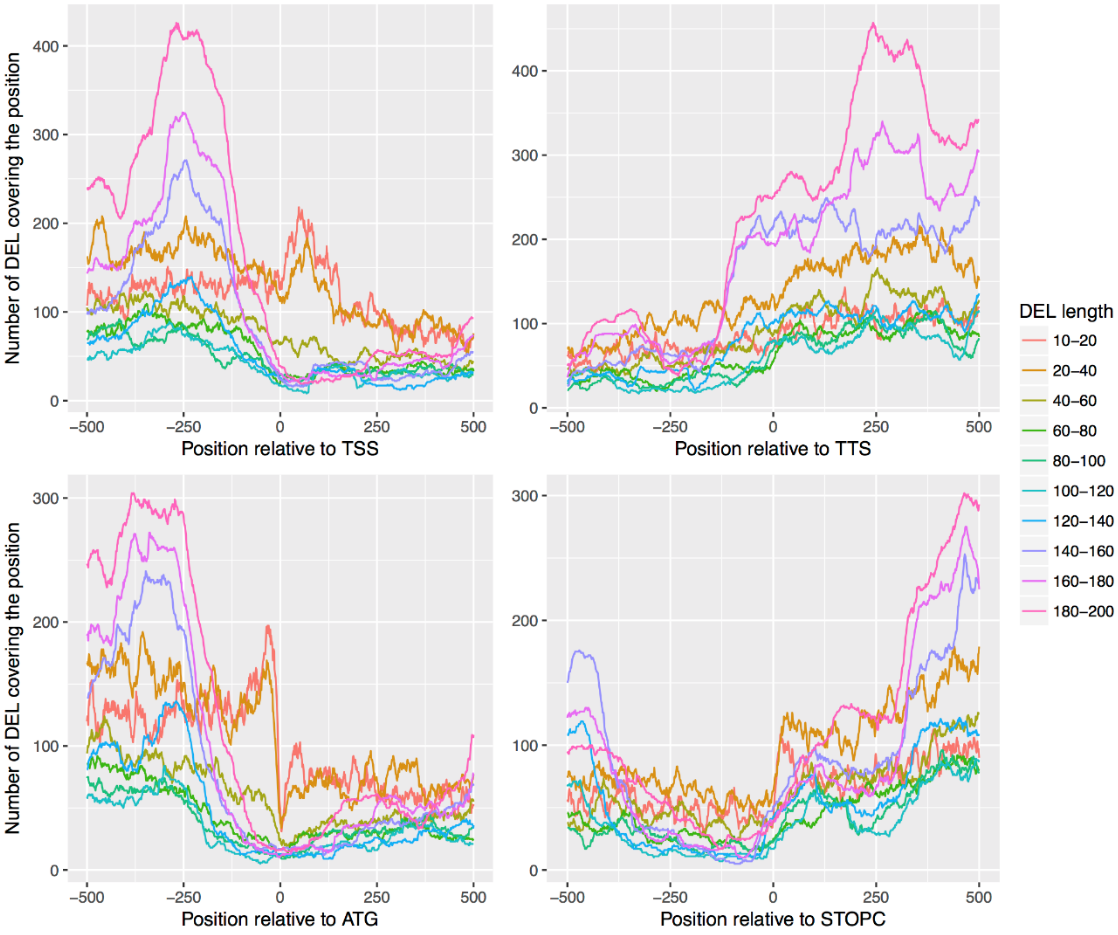

Figure S17. Dot plots for selected variants validated as true positives. a) Chromosome 10 11-kb deletion. **b**) Chromosome 10 218-bp tandem duplication and **c**) Chromosome 12 640-bp inversion. The blue cross lines mark the start position of the structural variant.

a

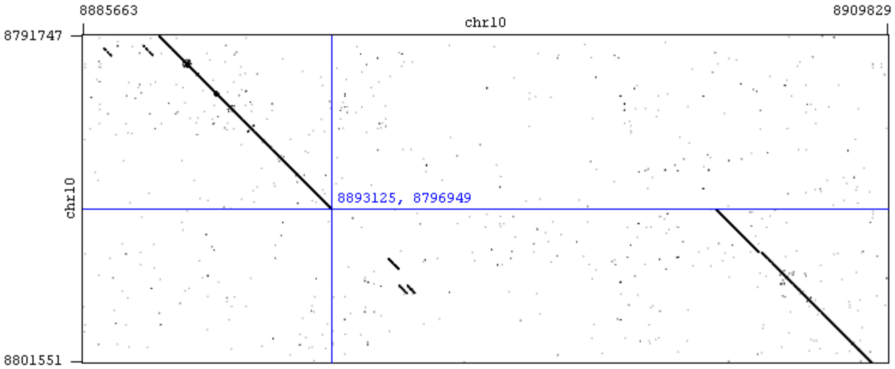

b

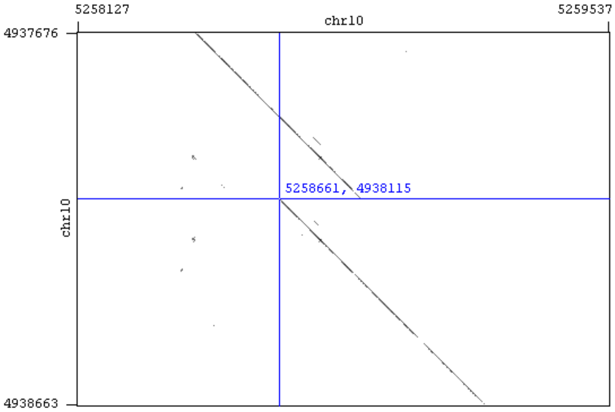

c

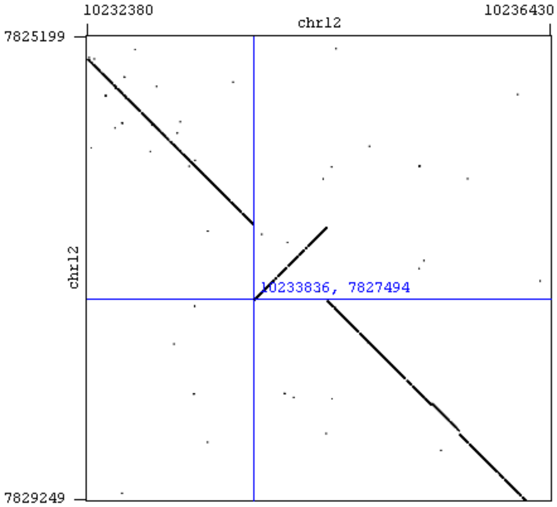

Figure S18. Assessment of copy number variation prediction. a) Read depth distribution for the Aus samples N22 and IRIS_313_11456 sequenced at about 12× average read depth. **b**) Number of copy number predictions and precision estimated by BLAST searches to the N22 assembly for five Aus samples sequenced at different average read depths.

a

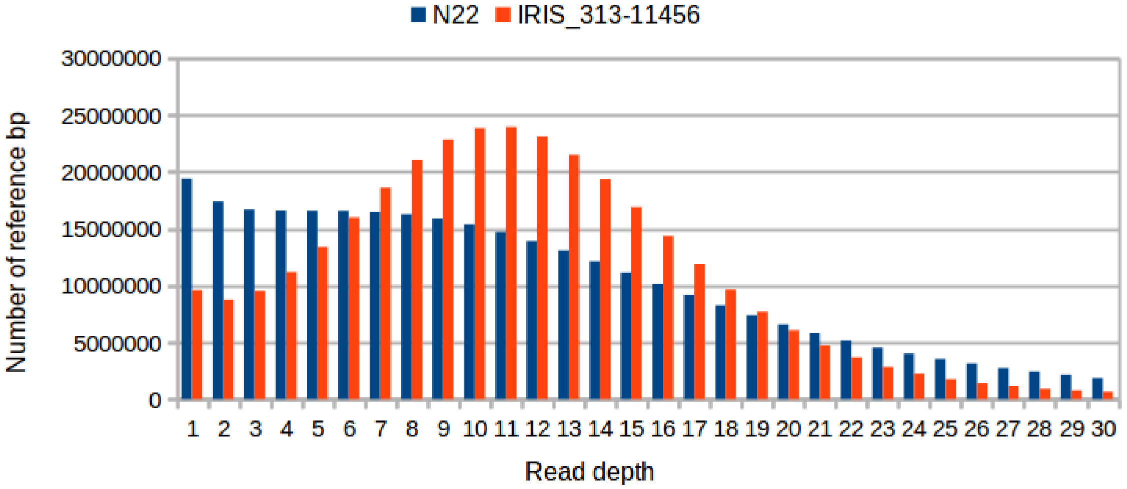

b

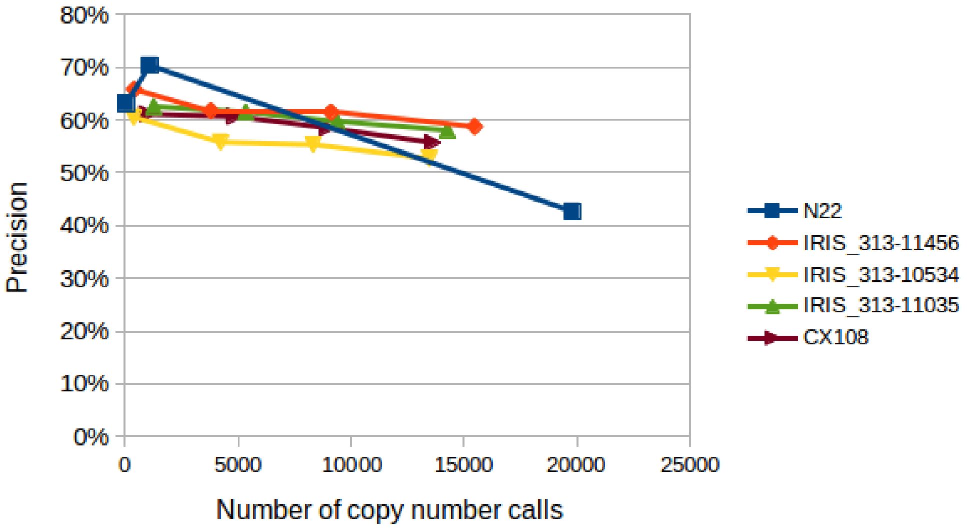

Figure S19. Comparison of deletion and insertion sequences to known or potentially active TEs. Each dot represents a cluster or an SV event. **a)** For deletions, extremely high or low- frequency TEs may indicate recent activity depending on whether they are retrotransposons (Class I; copy and paste) or DNA transposons (Class II; cut and paste). **b)** For insertions, cases with low allele frequency and high identity may indicate recent events. SV breakpoints do not always have precise breakpoints, hence lower sequence identity is expected especially for longer events. **c**) Insertional preference of INS events that matched active TE families.

a

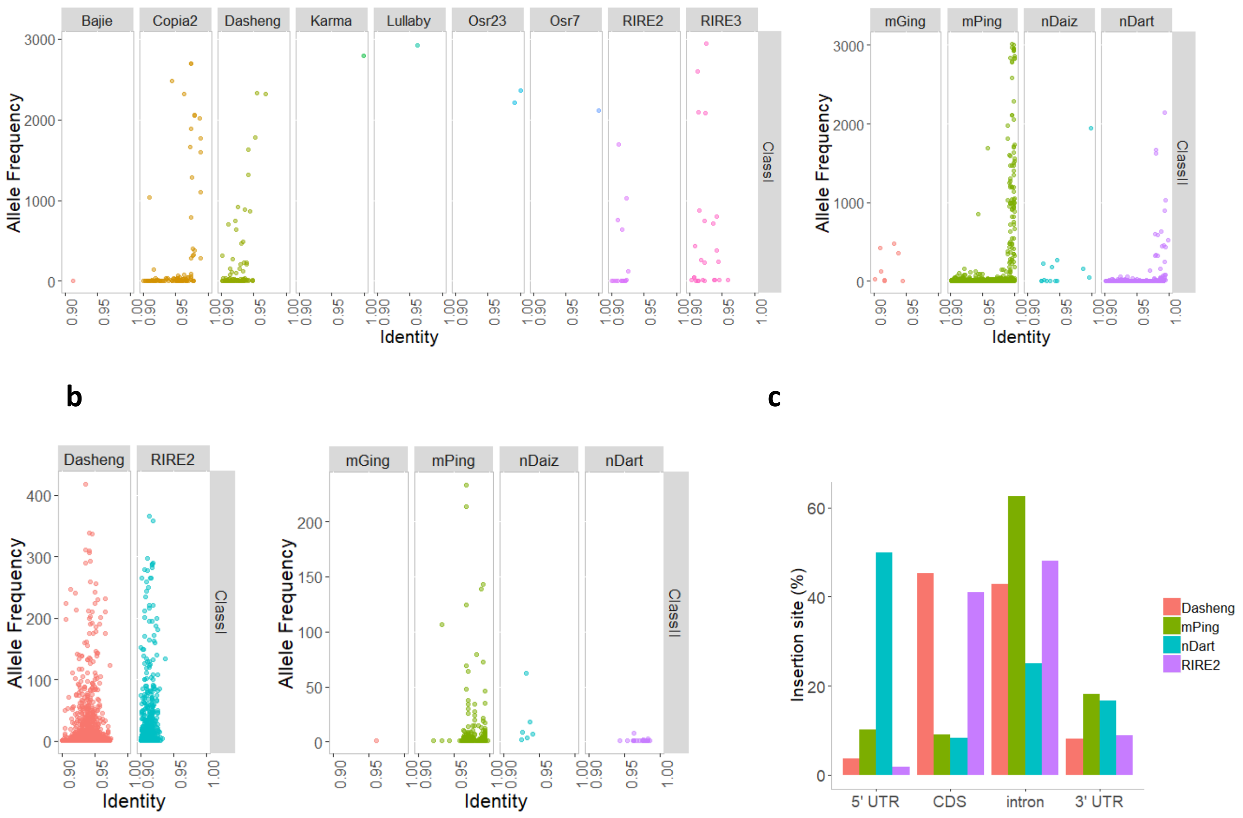

Supplemental Tables

Table S1. Transposable and repeat elements (size >50bp) among the structural variants.

Order	Superfamily	SV type (*no. observations / no. events*)			
		Deletion	Insertion*	Duplication	Inversion
Class I (Retrotransposons)					
LTR	Copia (RLC)	1,015,423 / 2,775	11,611 / 342	125,049 / 791	5,892 / 516
	Gypsy (RLG)	2,814,736 / 7,749	59,524 / 2,772	287,159 / 2,091	11,485 / 995
	ERV (RLE)	12,442 / 158	0	10 / 4	1,521/ 15
LINE	L1 (RIL)	187,686 / 2,105	58 / 5	1,738 / 18	221 / 42
SINE	- (RSU)	57,580 / 1,780	9,546 / 133	432 / 24	43 / 21
Class II (DNA transposons)					
TIR	Tc1-Mariner (DTT)	638,513 / 19,293	92,620 / 2,248	3,765 / 74	7,269 / 2,271
	hAT (DTA)	601,502 / 8,224	28,791 / 661	2,052 / 32	1,326 / 378
	Mutator (DTM)	1,445,375 / 16,853	106,758 / 2,699	1,880 / 67	16,362 / 1,455
	Harbinger (DTH)	1,864,512 / 36,689	305,963 / 6,899	2,358 / 91	5,033 / 1,482
	CACTA (DTC)	620,698 / 2,181	5,232 / 334	1032 / 41	1,133 / 129
	Micropon (DTN)	28,803 / 1,716	81 / 14	3 / 1	3 / 3
	MITE (DTI)	186,273 / 3,065	0	295 / 44	2,917 / 415
Helitron	Helitron (DHH)	316,409 / 2,155	1,093 / 48	2,227 / 73	890 / 98
Structural Repeat					
	Tandem repeats (SRR)	0	2 / 1	7 / 2	0
	Centromeric repeats (SRC)	1,287 / 81	3 / 3	1,334 / 71	266 / 48
	Telomeric repeats (SRT)	123 / 29	0	143 / 32	13 / 5
	Satellite (SRS)	0	0	0	0
	Simple repeats (SSS)	85,653 / 1,979	0	503 / 21	194 / 55
	Low Complexity (SSL)	1,776 / 151	0	90 / 8	50 / 18
	**Total**	9,878,791 / 106,983	621,282 / 16,159	430,077 / 3,485	54,618 / 7,946

* MetaSV insertions detected in 562 high-coverage samples

Table S2. Strategies and SV types supported by each caller.

Caller	Paired End	Split read	Read depth	*de novo* Assembly	Variant Types*
Delly	✔	✔			DEL,INS,DUP,INV,TRA
GROM	✔	✔	✔		DEL,INS,DUP,INV,TRA
GROM-RD			✔		DEL,DUP
Lumpy	✔	✔			DEL,INS,DUP,INV
MetaSV	✔	✔	✔	✔	DEL,INS
MindTheGap				✔	INS
NGSep	✔	✔	✔		DEL,DUP,INS,INV
Pindel	✔	✔			DEL,INS,DUP,INV
Scalpel				✔	DEL,INS,DUP,INV
Socrates		✔			DEL,INS,DUP,INV,TRA

***** DEL – deletion, INS – insertion, DUP – duplication, INV – inversion, TRA - translocation

Table S3. Summary of structural variants (SV) identified by NGSEP on the complete dataset combining read depth (RD), read pair (RP) and split read (SR) approaches.

Event	Analysis	Total events (Millions)	Average events per sample
Deletion	RD CNVnator	23.18	7,741.02
Deletion	RD EWT	29.91	9,985.46
Deletion	RP+SR	55.12	18,405.20
Duplication	RD CNVnator	18.18	6,070.59
Duplication	RD EWT	30.28	10,111.79

Table S4. Manually validated SVs using N22 (CX368) reference genome.

	Number of validated SV	T	FP	PT
Deletion	100	70	14	16 [a]
Duplication	40	16	16	8 [b]
Inversion	20	5	15 [c]	0 [c]

[a] Partially True: Inaccurate breakpoint, longer or complex event
[b] Transposable element
[c] Includes inverted repeats and palindromes

Supplemental Methods

Structural variant discovery

Structural variants can be classified into the following types: deletions, insertions, duplications (tandem and interspersed), inversions, and translocations, and there are five general strategies to detect SVs based on analysis of data from high throughput DNA sequencing technologies: paired-end mapping (RP), split-read mapping (SR), read depth (RD), *de novo* assembly (AS), and a combination of the above approaches (CB). Each of these strategies have different strengths and weaknesses in detection, depending on variant type, sequence length and reference genome quality; hence, applying complementary methods and combining results can overcome some of the limitations inherent to the different approaches (Alkan et al. 2011). To improve performance and reduce false detections, we combined multiple algorithms into a pipeline for SV discovery.

Breakdancer (Chen et al. 2009) and GASV (Sindi et al. 2009) are two examples of RP algorithms that analyze relatively discordantly aligned paired reads, while an SR method called Socrates (Schröder et al. 2014) considers cases of gapped or broken read alignments to the reference genome. Both RP and SR methods can be used to discover all classes of SVs, but SR has the advantage of single-nucleotide resolution (Alkan et al. 2011). RD methods like GROM-RD (Smith et al. 2015) and CNVnator (Abyzov et al. 2011) involve counting reads in windows and segmenting counts; although these RD approaches are able to detect both tandem and interspersed duplications, they only detect losses (deletions) and gains (duplication), rely on good read coverage, and have poor breakpoint resolution (Francia et al. 2015; Tattini et al. 2015). On the other hand, some SV callers that combine multiple signals such as GROM (Smith et al. 2017), Pindel (Ye et al. 2009), Delly (Rausch et al. 2012), NGSEP (Duitama et al. 2014) and Lumpy (Layer et al. 2014) provide high precision and sensitivity for insertions, deletions, inversions and tandem duplications but not all are able to identify interspersed duplications.

Most of the previous methods mentioned rely on read alignment to a single reference genome; hence, for rice, long novel insertion events relative to the Nipponbare reference genome (IRGSP 1.0) cannot be detected (Schatz et al. 2014). The extensive diversity of rice landraces and varieties exemplified by deep population structure and differences in genome sizes will also impact the ability to detect SVs accurately. *De novo* assembly methods such as Scalpel (Narzisi et al. 2015) and ScanIndel (Yang et al. 2015), assemble reads from suspected breakpoints into contigs and then align these contigs to a reference genome to determine the exact boundaries. AS methods can detect all types of SVs at nucleotide resolution but are computationally intensive and not applicable to low-coverage samples. MindTheGap (Rizk et al. 2014), a variant of AS, implements the Bloom filter, a probabilistic space-efficient data structure, to create a probabilistic de Bruijn graph and requires less memory compared to other AS methods. To further enhance the efficiency of structural variant discovery, newer solutions like SVMerge (Wong et al. 2010) and MetaSV (Mohiyuddin et al. 2015) combine predictions from multiple callers, filter pooled results and refine breakpoints using local assembly. Apart from the more common methods presented above, another tool named forests (Michaelson and Sebat 2012) implements machine learning trained with experimentally-validated structural variant calls, but is currently limited to the human genome. Supplemental Table S2 provides the summary of strategies used and the supported SV types per caller.

Comparison of structural variant finding programs

To identify a set of SV callers to be integrated into our discovery pipeline, we benchmarked ten callers (i.e. Pindel, Delly, GROM, NGSEP, GROM-RD, Lumpy, Socrates, MindTheGap, MetaSV, Scalpel) in terms of precision and sensitivity (recall) of detecting different types and lengths of simulated SVs. Shown in **Figure S1**, Pindel, Delly and GROM have relatively good F1-scores for detection of deletions and tandem duplications. F1-score computed as $\frac{2(recall*precision)}{(recall+precision)}$ is the harmonic average of computed precision and recall. For inversions, Pindel and Lumpy gave the best performance, while Delly and GROM discovered

very few events in the first two bins. Scalpel always produced very precise predictions, but was not as sensitive as the others. Lumpy performed best for inversions with low sensitivity for small deletions and tandem duplications.

Since long insertions with precise breakpoints and sequences are detected more accurately by assembly-based tools, MetaSV, MindTheGap, and Scalpel were also evaluated. ScanIndel was eliminated from the list due to errors when running larger bins. Only MindTheGap had >50% sensitivity on bin A while MetaSV had the highest sensitivity for all the other bins. Pindel had the highest sensitivity among non-assembly-based tools, but it could not determine sequences for large insertions. Currently, it is still challenging to assess SV callers to run on rice genomes due to the absence of a gold standard list of validated structural variants.

Comparison with GATK

We compared the insertion and deletion datasets with the indels deposited in the SNP-Seek database (Mansueto et al. 2017) and found that setting 50 bp as the minimum SV size in our pipeline discarded variants of sizes below 50 bp that the GATK-UG algorithm failed to detect. Reducing the minimum length to 10 bp for DEL and 5 bp for INS allowed the pipeline to report calls that GATK-UG missed due to its algorithmic limitation(s). **Figure S1** shows the range of deletion and insertion sizes GATK-UG and our new pipeline were able to detect. From this figure, we see that GATK-UG can detect most deletions less than 30 bp (also see **Figure S1**) and most insertions shorter than 20 bp. For greater sizes, we need to apply specialized methods designed for detecting large SVs.

SV detection and genotyping of CNVs with NGSEP

Copy number variants (CNVs) and large deletions were identified independently in the 3K RG samples for both read depth (RD) and read pair/split read (RP+SR) analyses in NGSEP using default parameters. The

RD analysis used both the CNVnator algorithm and the EWT algorithm. Between 13,000 and 20,000 events were detected on average per sample using each approach (**Table S3**).

The distribution of lengths for deletions and duplications called by each approach shows that the CNVnator algorithm tends to call both deletions and duplications with average lengths of around 10 kbp, whereas the EWT algorithm and the RP+SR analysis call mainly events of lengths below 2 Kbp (**Figure S13a**). The events called by CNVnator (both deletions and duplications) cover on average 60Mbp of the genome for each sample, whereas the events called by EWT cover on average 10Mbp and always less than 40Mbp (**Figure S13b**). Interestingly, the amount of the genome covered by deletions called by the RP+SR analysis is on average 80 Mbp of the genome. This is mainly explained by the tail of long events called with this analysis (Last bar of **Figure S13a**).

Since the RP+SR approach is also implemented by other tools compared in the analysis, and that Pindel and Delly reported better accuracy using simulated data, the rest of this analysis focused on CNVs predicted with the CNVnator algorithm. The distribution of lengths and genome coverage shown in **Figure S13** suggests that this analysis provides deletions and duplications that cannot be identified by other approaches, complementing the results provided by the other tools. It is well known that the accuracy of the RD analysis is mainly affected by the average read depth at which each sample is sequenced, the distribution of reads across the genome, and the amount of repetitive content within each particular region (Teo et al. 2012; Duitama et al. 2014). To account for the first two issues, a subset of 938 samples was selected if the average read depth was 15× and if the total genome covered by duplications was below 100 Mb. The latter filter assumes that samples for which CNV predictions cover a larger percentage of the genome are likely to contain more false positive calls due to an overall non-uniform distribution of reads. Calls from these samples were merged into a consolidated set of 365,761 regions affected by CNVs using a heuristic procedure described in Lobaton et al. (2018) for a similar study in common bean . The repetitive content of each of these CNV regions was calculated and annotated to allow filtering of regions using

different thresholds based on the percentage of repetitive content depending on the particular downstream analysis of these CNVs.

Copy number for each CNV region for each of the 3K RG samples was based on the read-depth within the region as compared to the average read-depth across the genome for a particular sample taking into account its variance (see Lobaton et al. (2018) for details). This procedure led to a matrix of predictions of copy number, which we term CNV genotype calls, having as many rows as CNVs and as many columns as analyzed samples. A dataset of 669 million genotype calls with minimum genotyping quality score of 10 was assembled, having a percentage of missing data of 39.5%. If only the 938 samples selected for the merging step are taken into account, the number of genotype calls reduces to 243 million, with a percentage of missing data of 29.1%. This dataset represents a raw catalog of copy number variation events that, similar to the database of SNPs (Mansueto et al. 2017) can be filtered in different ways depending on the expected level of precision and sensitivity and on the desired downstream analysis. For example, since the confounding effects produced by misalignments around repetitive regions is the main source of false positive predictions, application of stringent filtering removed regions that overlap by even 1bp with repetitive regions and reduced the dataset to 5,351 CNV regions and 8.7 million genotype calls over the 3K RG samples having a percentage of missing data of 46.3%. Within the samples selected for the merging step, the number of genotype calls becomes 3.2 million (36.4% missing data).

Validation of CNVs

We performed two different approaches for validation of CNVs. First, we used predictions of copy number variation as alleles of genetic markers and performed clustering of samples provided by the structure software (Pritchard et al. 2000) on different datasets obtained by applying filters of number of samples genotyped, intersection with repeat regions and quality score of the SV calls (see results in the main text).

Second, we tried to validate the predictions of copy number of the sample N22 by performing BLAST searches in the recently published *de novo* assembly of this sample and checking if the number of copies found in the assembly corresponds with the predictions copy number based on RD. Unfortunately, the read depth distribution for this sample deviated severely from a normal distribution (**Figure S19a**). We followed this procedure with other Aus samples assuming that the Aus samples would share a common core of structural variation relative to Nipponbare. The precision obtained with the raw dataset of calls was about 20%. However, we noticed that most false positive calls were those with predictions of copy number equal to 1 (heterozygous deletion) or 3 (heterozygous duplication). Hence, we decided to filter out these calls and then remove CNVs that do not have predictions of copy number below 1 or above 3 on the 938 selected samples. This filter produced the final dataset of CNVs for this study having 207,927 CNVs. **Figure S19b** shows that the estimated precision in this dataset increases close to 60%, even taking into account that the assembly used for comparison corresponds to a sample different from the tested samples. Further filtering based on quality score only reduced recall without increasing precision.

References

Abyzov A, Urban AE, Snyder M, Gerstein M. 2011. CNVnator: An approach to discover, genotype, and characterize typical and atypical CNVs from family and population genome sequencing. *Genome Research* **21**: 974-984.

Alkan C, Coe BP, Eichler EE. 2011. Genome structural variation discovery and genotyping. *Nature reviews Genetics* **12**: 363-376.

Chen K, Wallis JW, McLellan MD, Larson DE, Kalicki JM, Pohl CS, McGrath SD, Wendl MC, Zhang Q, Locke DP et al. 2009. Breakdancer: an algorithm for high resolution mapping of genomic structural variation. *Nature Methods* **6**: 677-681.

Duitama J, Quintero JC, Cruz DF, Quintero C, Hubmann G, Foulquié-Moreno MR, Verstrepen KJ, Thevelein JM, Tohme J. 2014. NGSep: An integrated framework for discovery and genotyping of genomic variants from high-throughput sequencing experiments. *Nucleic acids research* **42**: e44-e44.

Francia E, Pecchioni N, Policriti A, Scalabrin S. 2015. CNV and Structural Variation in Plants: Prospects of NGS Approaches. doi:10.1007/978-3-319-17157-9, pp. 211-232. Springer International Publishing.

Layer RM, Chiang C, Quinlan AR, Hall IM. 2014. LUMPY: A probabilistic framework for structural variant discovery. *Genome biology* **15**: R84-R84.

Lobaton JD, Miller T, Gil J, Ariza D, de la Hoz JF, Soler A, Beebe S, Duitama J, Gepts P, Raatz B. 2018. Resequencing of Common Bean Identifies Regions of Inter-Gene Pool Introgression and Provides Comprehensive Resources for Molecular Breeding. *Plant Genome* **11**.

Mansueto L, Fuentes RR, Borja FN, Detras J, Abriol-Santos JM, Chebotarov D, Sanciangco M, Palis K, Copetti D, Poliakov A et al. 2017. Rice SNP-seek database update: new SNPs, indels, and queries. *Nucleic acids research* **45**: D1075-D1081.

Michaelson JJ, Sebat J. 2012. forestSV: structural variant discovery through statistical learning. **9**: 819-821.

Mohiyuddin M, Mu JC, Li J, Bani Asadi N, Gerstein MB, Abyzov A, Wong WH, Lam HYK. 2015. MetaSV: an accurate and integrative structural-variant caller for next generation sequencing. *Bioinformatics (Oxford, England)* doi:10.1093/bioinformatics/btv204: 1-4.

Narzisi G, Rawe JAO, Iossifov I, Lee Y-h. 2015. Accurate de novo and transmitted indel detection in exome-capture data using microassembly. *Nature Methods* **11**: 1033-1036.

Pritchard JK, Stephens M, Donnelly P. 2000. Inference of population structure using multilocus genotype data. *Genetics* **155**: 945-959.

Rausch T, Zichner T, Schlattl A, Stütz AM, Benes V, Korbel JO. 2012. DELLY: structural variant discovery by integrated paired-end and split-read analysis. *Bioinformatics (Oxford, England)* **28**: i333-i339.

Rizk G, Gouin A, Chikhi R, Lemaitre C. 2014. MindTheGap : integrated detection and assembly of short and long insertions. *Bioinformatics*: 1-7.

Schatz MC, Maron LG, Stein JC, Wences AH, Gurtowski J, Biggers E, Lee H, Kramer M, Antoniou E, Ghiban E et al. 2014. Whole genome de novo assemblies of three divergent strains of rice, Oryza sativa, document novel gene space of aus and indica. *Genome Biology* **15**: 506-506.

Schröder J, Hsu A, Boyle SE, Macintyre G, Cmero M, Tothill RW, Johnstone RW, Shackleton M, Papenfuss AT. 2014. Socrates: identification of genomic rearrangements in tumour genomes by re-aligning soft clipped reads. *Bioinformatics (Oxford, England)* doi:10.1093/bioinformatics/btt767: 1-9.

Sindi S, Helman E, Bashir A, Raphael BJ. 2009. A geometric approach for classification and comparison of structural variants. *Bioinformatics* **25**: 222-230.

Smith SD, Kawash JK, Grigoriev A. 2015. GROM-RD: resolving genomic biases to improve read depth detection of copy number variants. *PeerJ* **3**: e836-e836.

Smith SD, Kawash JK, Grigoriev A. 2017. Lightning-fast genome variant detection with GROM. *GigaScience* **6**: 1-7.

Tattini L, D'Aurizio R, Magi A. 2015. Detection of Genomic Structural Variants from Next-Generation Sequencing Data. *Frontiers in bioengineering and biotechnology* **3**: 92-92.

Teo SM, Pawitan Y, Ku CS, Chia KS, Salim A. 2012. Statistical challenges associated with detecting copy number variations with next-generation sequencing. *Bioinformatics* **28**: 2711-2718.

Wong K, Keane TM, Stalker J, Adams DJ. 2010. Enhanced structural variant and breakpoint detection using SVMerge by integration of multiple detection methods and local assembly. *Genome biology* **11**: R128-R128.

Yang R, Nelson AC, Henzler C, Thyagarajan B, Silverstein KaT. 2015. ScanIndel: a hybrid framework for indel detection via gapped alignment, split reads and de novo assembly. *Genome medicine* **7**: 127-127.

Ye K, Schulz MH, Long Q, Apweiler R, Ning Z. 2009. Pindel: a pattern growth approach to detect break points of large deletions and medium sized insertions from paired-end short reads. *Bioinformatics (Oxford, England)* **25**: 2865-2871.
